# Supplementary material for: Facet-selective growth of halide perovskite/2D semiconductor van der Waals heterostructures for improved optical gain and lasing
Source: Nat Commun. 2024 Jun 28;15:5484. doi: 10.1038/s41467-024-49364-0 (PMC11213932; doi:10.1038/s41467-024-49364-0)
Supplement: Supplementary file 1 — Supplementary Information [file 41467_2024_49364_MOESM1_ESM.pdf]

Supplementary Information for

Facet-selective growth of halide perovskite/2D semiconductor van der Waals heterostructures for improved optical gain and lasing

Liqiang Zhang[1,†], Yiliu Wang[2,†], Anshi Chu[2,†], Zhengwei Zhang[3], Miaomiao Liu[1] Xiaohua Shen[1], Bailin Li[1], Xu Li[2], Chen Yi[2], Rong Song[1], Yingying Liu[1], Xiujuan Zhuang[4], Xidong Duan[1*]

1 Hunan Provincial Key Laboratory of Two-Dimensional Materials,
State Key Laboratory for Chemo/Biosensing and Chemometrics,
College of Chemistry and Chemical Engineering,
Hunan University, Changsha, P. R. China
2 Key Laboratory for Micro-Nano Optoelectronic Devices of Ministry of Education, School of Physics and Electronics, Hunan University, Changsha 410082, China.
3 Hunan Key Laboratory of Nanophotonics and Devices, School of Physics, Central South University, Changsha, Hunan, P. R. China.
4 College of Semiconductors (College of Integrated Circuits), Hunan University, Changsha, Hunan, P. R. China

† These authors contributed equally: Liqiang Zhang, Yiliu Wang, Anshi Chu
[*]Corresponding author: Xidong Duan (xidongduan@hnu.edu.cn)

Supplementary Fig. 1. Reactivity tolerance in direct epitaxy of halide perovskite/2D semiconductor heterostructures

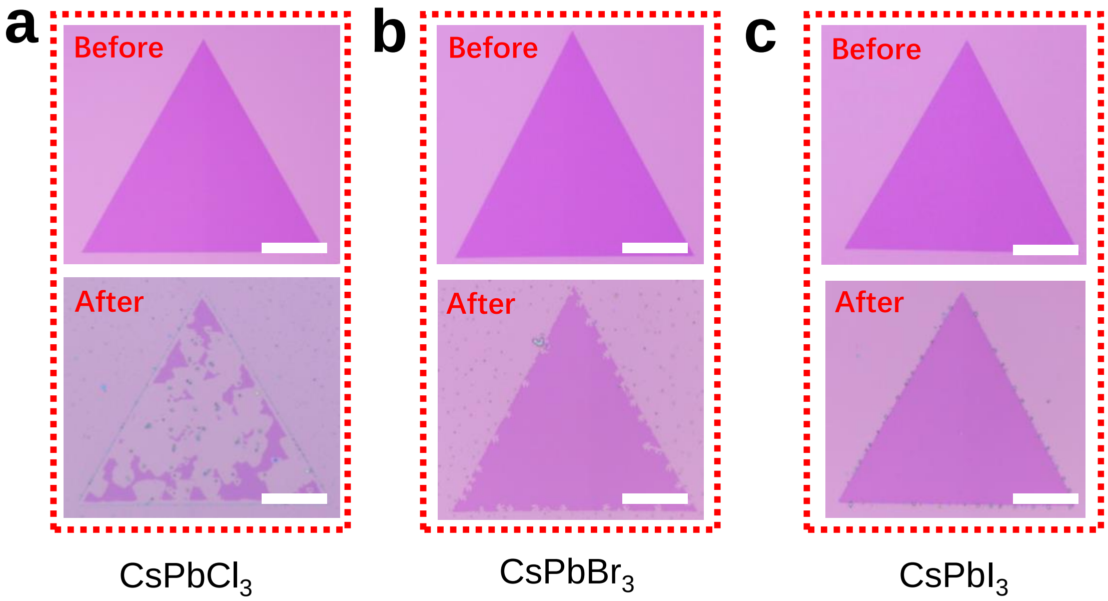

Supplementary Fig. 1. Etching effect of halide perovskite on monolayer WSe_2. a The $CsPbCl_3$ shows intensive etching effect on monolayer WSe_2, with most WSe_2 encroached. **b** The $CsPbBr_3$ shows moderate etching effect on monolayer WSe_2, with part eroded from edge to center. **c** The $CsPbI_3$ has no etching effect on monolayer WSe_2, with pristine structure well maintained. Scale bar, 30 μm.

Currently, direct epitaxy of halide perovskite on 2D semiconductors has been achieved remarkable advances and focused on solution-phase epitaxy, but the uncontrolled fast crystallization of ionic halide perovskites results in polycrystalline film, in which randomly distributed defects and grain boundary hamper the photonic applications. Vapor-phase van der Waals epitaxy is a unique way to flexibly control the crystallization of halide perovskite on 2D semiconductors, to form clean interface, thus establishing an ideal model system for examining the pristine behavior of crystal growth and optical gain property on single-particle level free of inter-crystal influence, but the fabrication of such prefect heterostructure fails to experimentally realize so far, the main obstacle comes from the chemical interaction between halide perovskites and 2D semiconductors. Taking WSe_2, a typical 2D transition metal dichalcogenide (TMD), as an example, the etching effect of halide perovskite clearly observed, as shown in Supplementary Fig. 1a-c.

From experimental observations, the triangle-shaped monolayer WSe_2 was etched by both $CsPbCl_3$ and $CsPbBr_3$, while the etching intensity of $CsPbCl_3$ was far more intensive than that of $CsPbBr_3$, reflected by most WSe_2 film disappeared after exposure to $CsPbCl_3$ but only part WSe_2 film removed after exposure to $CsPbBr_3$. Interestingly, the $CsPbI_3$ has no etching effect on monolayer WSe_2, with whole WSe_2 film sustained over growth during, suggesting that $CsPbI_3$ is expectedly friendly to WSe_2 and feasible to directly grow heterostructures, the sustainability and quality retention of monolayer

detailedly discussed below. On the whole, the etching intensity of halide perovskite towards monolayer WSe_2 follows the order: $CsPbCl_3$> $CsPbBr_3$>$CsPbI_3$, the reason for which may possible be attributed to their relative oxidation potentials of halide species (Cl>Br>I)[1]. This result occurs for other 2D TMDs (WS_2, MoS_2 and $MoSe_2$) because of their iso-structural nature and similar chemistry attributes. Meanwhile, we also find that the mixed halide perovskite $CsPbI_2Br$ is also well friendly to 2D TMDs possibly due to a small concentration of Br specie, which indicates the possibility of tailoring the property of heterostructure through compositional engineering.

Supplementary Fig. 2. General applicability of van der Waals epitaxy of halide perovskite/2D semiconductor heterostructures to different substrates

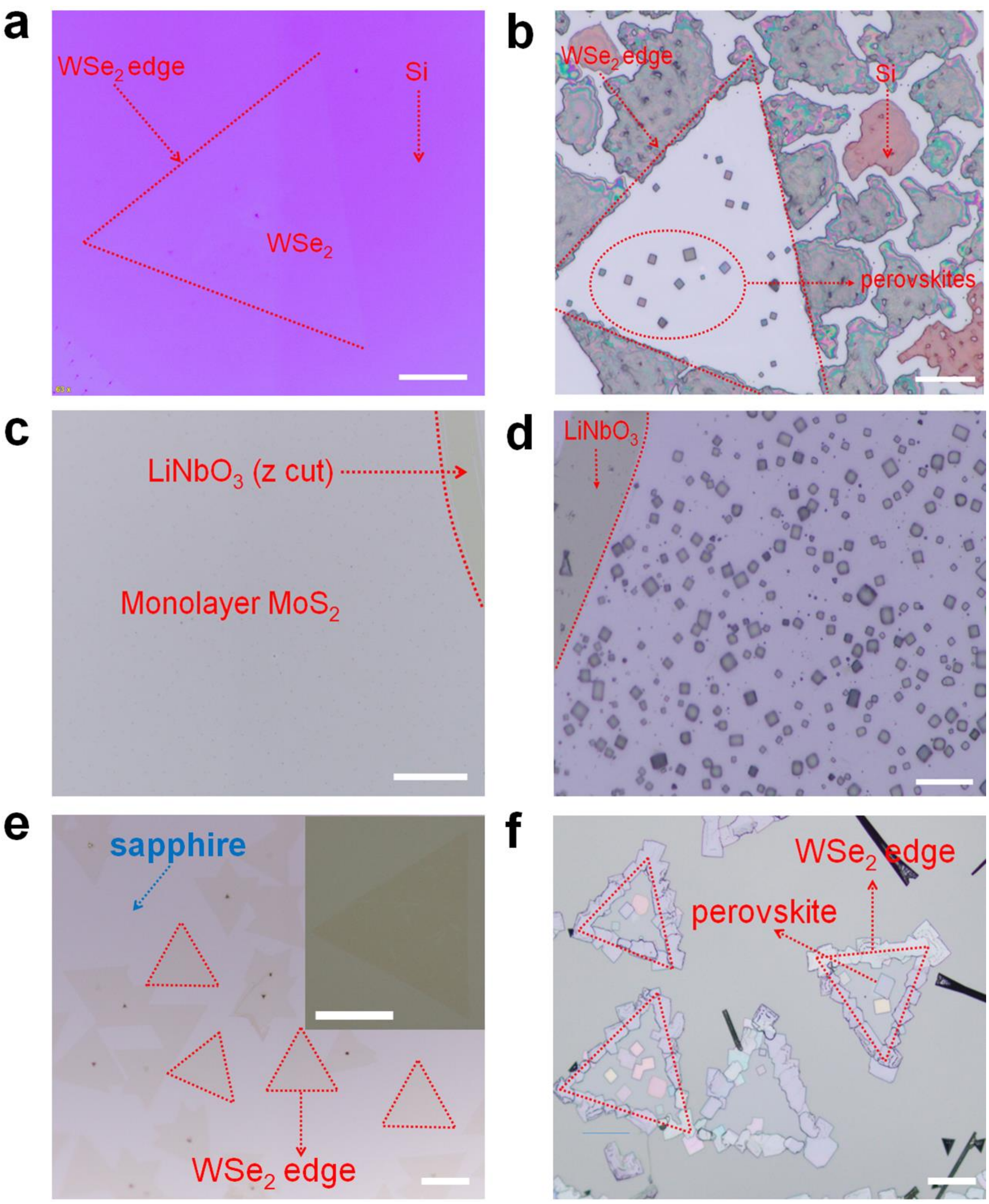

Supplementary Fig. 2. The general applicability of our direct van der Waals epitaxy of halide perovskite/2D semiconductor heterostructures to different

substrates. **a-b** Monolayer WSe_2 (triangle shape highlighted by red dotted lines) on Si substrate and $CsPbI_2Br/WSe_2$ heterostructures grown on Si substrate using direct van der Waals epitaxy, scale bar, 50 μm. **c-d** Monolayer MoS_2 film on $LiNbO_3$ substrate and $CsPbI_2Br/MoS_2$ heterostructures grown on $LiNbO_3$ substrate using direct van der Waals epitaxy. Scale bars for 100 μm (in (**c**) and 50 μm in (**d**). **e** Monolayer WSe_2 on double-polished sapphire substrate, scale bar, 50 μm, inset is a zoom-in optical image of single monolayer WSe_2 domain, scale bar, 40 μm. **f** optical image of $CsPbI_2Br/WSe_2$ heterostructures grown on sapphire substrate, scale bar, 50 μm.

The monolithically integrated halide perovskite/2D semiconductor heterostructures on selected substrates are shown below. The growth methodology and parameters are identical to van der Waals epitaxy used for SiO_2/Si substrate. Supplementary Fig. 2a displays the monolayer WSe_2 grown on Si substrate. Due to the subtle color contrast between monolayer WSe_2 and Si substrate surface, we adjust the color contrast of optical microscopy to visualize the monolayer WSe_2, which is marked by red dotted lines. The deposited WSe_2 on Si substrate is typical triangular single crystal domain. The resultant $CsPbI_2Br/WSe_2$ heterostructures are shown in Supplementary Fig. 2b, where rectangle halide perovskites are only epitaxially grown on monolayer WSe_2 surface and are well aligned with the edges of monolayer WSe_2. The pure Si surface is filled with irregular polycrystalline films and/or deposited precursor particles. This selective and oriented growth is consistent with the growth habit observed on SiO_2/Si substrate. Supplementary Fig. 2c-d exhibit large-area monolayer MoS_2 film on $LiNbO_3$ (z cut) and their $CsPbI_2Br/MoS_2$ heterostructures. It is obvious that the rectangle perovskite single crystals are preferentially grown on monolayer MoS_2 surface, while pyramid-shaped perovskites only nucleated and grew on pure $LiNbO_3$ surface. This also strongly prove the robustness and scalability of van der Waals epitaxial strategy. Supplementary Fig. 2e is optical image of monolayer WSe_2 on double-polished sapphire substrate, the inset is a zoom-in optical image of single monolayer WSe_2 domain, which is manifested as a triangular single crystal domain. Supplementary Fig. 2f shows $CsPbI_2Br/MoS_2$ heterostructures grown by van der Waals epitaxy. The epitaxial perovskite single crystals all are selectively grown and aligned on monolayer WSe_2 surface. The successful epitaxial growth of halide perovskite/2D semiconductor heterostructures on Si, $LiNbO_3$ and sapphire (Al_2O_3) substrates robustly demonstrate the general applicability to different substrates.

Supplementary Fig. 3. Raman and photoluminescence spectra of 2D semiconductors and heterostructure

The Raman spectrums of all 2D semiconductors synthesized here show two prominent characteristic peaks (Supplementary Fig. 3a, b): 249 cm^{-1} (E^1_{2g}) and 259 cm^{-1} (A_{1g}) for WSe_2, 256 cm^{-1} (E^1_{2g}) and 419 cm^{-1} (A_{1g}) for WS_2, 384 cm^{-1} (E^1_{2g}) and 403 cm^{-1} (A_{1g}) for MoS_2, 240 cm^{-1} (A_{1g}) and 285 cm^{-1} (E^1_{2g}) for $MoSe_2$, being prefect agreement with the previous reports. Accordingly, the sharp photoluminescence (PL) signals of monolayer semiconductors are centered at 776 nm, 632 nm, 681 nm and 814 nm for WSe_2, WS_2, MoS_2 and $MoSe_2$, respectively (Supplementary Fig. 3d, e), showing

monolayer nature and highly being compatible with the prior reports[2,3]. The Raman spectra of SnS_2/WSe_2 heterostructure is shown in Supplementary Fig. 3c, where a new weak peak located at 314 cm^{-1} is clearly observed, originating from the monolayer SnS_2. The PL spectra of that shows obvious quenching compared with monolayer WSe_2 (Supplementary Fig. 3f), stemming from the fast interlay charge transfer[4].

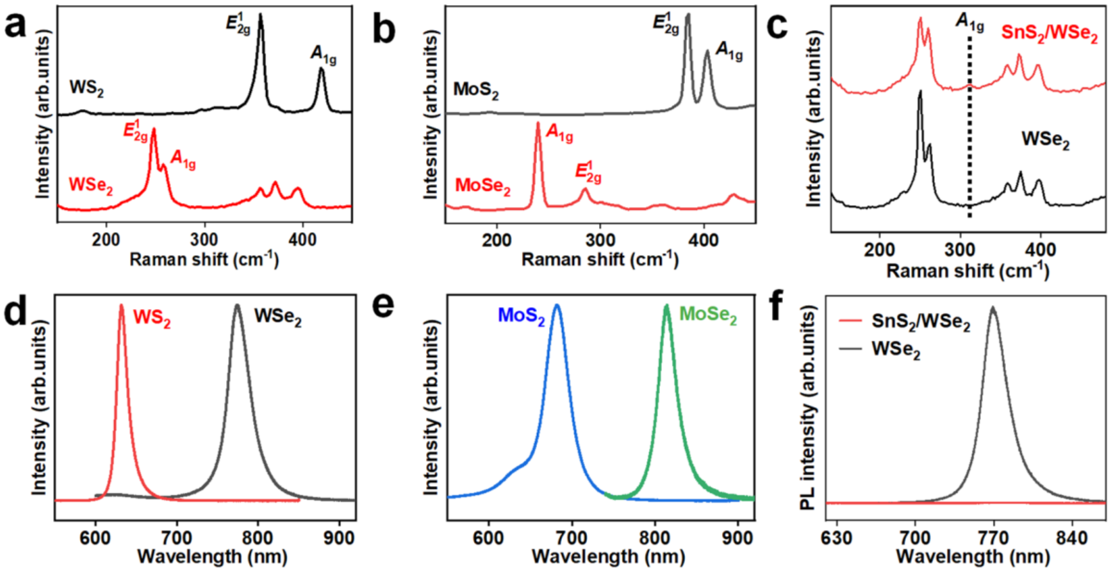

Supplementary Fig. 3. Raman and photoluminescence spectra of 2D single semiconductors and heterostructures. a-c Raman spectral of monolayer WSe_2, WS_2, MoS_2, $MoSe_2$ and SnS_2/WSe_2. **d-f** photoluminescence spectral of monolayer WSe_2, WS_2, MoS_2, $MoSe_2$ and SnS_2/WSe_2.

Supplementary Fig. 4. The monolayer nature and stability of WSe_2

The monolayer nature of WSe_2 obtained were confirmed by Atomic Force Microscope (AFM) image and phase image (Supplementary Fig. 4a-b). The height profile shows an about 0.82 nm thickness, which is in agreement with the monolayer feature of WSe_2. The phase image give a direct evidence that the surface of monolayer WSe_2 used here is highly clean, which is critically important to epitaxially grow high-quality $CsPbI_3$ perovskite with specific facet-orientation selectivity. The absence of Raman peak at 308 cm^{-1} and strong emission of PL also demonstrate the single layer nature of WSe_2 used here. To elucidate the effect of $CsPbI_3$ epitaxy on WSe_2 monolayer, the Raman mapping/spectra and PL mapping/spectra were collected. The Supplementary Fig. 4d and g present the resulting optical images, where the square-shaped crystal is $CsPbI_3$ and the neighboring region in pink color is WSe_2 monolayer. It should be note that the laser intensity should be controlled at relatively lower level during the tests in order to reduce the extensive damage of perovskite, which may influence the actuality and uniformity of Raman/PL images. The Raman mapping (Supplementary Fig. 4e) shows uniform signal at 249 cm^{-1} expect for the perovskite crystal region. The absence of Raman signal in the $CsPbI_3/WSe_2$ heterostructure region originates from the strong absorption of upper $CsPbI_3$ crystal, which greatly dissipates the energy from excitation laser. Based on the Raman spectra of WSe_2 obtained at different conditions (Supplementary Fig. 4f), WSe_2 before $CsPbI_3$ epitaxy and after

$CsPbI_3$ epitaxy as well as WSe_2 underneath the $CsPbI_3$ crystal (after washing away $CsPbI_3$ with acetone), the characteristic Raman peaks at 249 cm^{-1} and 259 cm^{-1} are clearly observed, which are assigned to E^{1}_{2g} and A_{1g} resonance modes, respectively. Other high-energy peaks at 360 cm^{-1}, 374 cm^{-1} and 397 cm^{-1} attributed to the $2E_{1g}$, A_{1g}+LA and A_{1g}-LA modes, respectively, corresponding to the second order and combinational modes[5]. Moreover, all the characteristic peaks show invariant position, manifesting good sustainability of monolayer WSe_2 during epitaxial growth process. Analogously, the PL mapping image and PL spectra also show similar feature (Supplementary Fig. 4h and i). The weak PL emission in $CsPbI_3/WSe_2$ heterostructure region is attributed to PL quenching effect from interfacial charge transfer. The PL spectra at all conditions experienced no obvious peak position shift (Supplementary Fig. 4i), only the peak symmetry is upward a little, possibly due to the altered WSe_2-substrate interactions from annealing during epitaxial growth process and decoupling effect after being exposed to acetone washing[6]. These experimental observations demonstrated that the monolayer WSe_2 survived and no defect generation/healing of WSe_2 monolayer at the whole $CsPbI_3$ epitaxial growth process.

The generation/healing of WSe_2 defects during epitaxial growth is most unlikely to occur. The qualitative and quantitative analysis are shown below. Qualitatively speaking, a thorough literature survey demonstrates that the substantial defects, strain and doping effect introduced from halide interaction mainly take place at temperature range of more than 700 ℃[7-9]. Our growth temperature (500 ℃) is far lower than that the reported to be more than 700℃, thus ruling out the possibility of generation of WSe_2 defects in our epitaxial growth process. Additionally, the halide species could preferentially passivate the edges of the 2D WSe_2 monolayer due to their prominent dangling bond states. This edge passivation effect may relax in-plane strains to suppress defect generation[10].

The quality variation of monolayer WSe_2 were further quantitative monitored by time-resolved photoluminescence (TRPL), the result of which is shown in Supplementary Fig. 4c. We analyzed the fast (τ_1) and slow (τ_2) decay components of monolayer WSe_2 before and after $CsPbI_3$ epitaxy using the bi-exponential decay function $y = A_1\exp(-t/\tau_1) + A_2\exp(-t/\tau_2)$, where A is the amplitude components and t is the time constant. The fitting parameters are listed in Supplementary Table 1, from which we can see that before and after $CsPbI_3$ epitaxy, all important parameters almost kept unchanged, indicating the excellent quality retention.

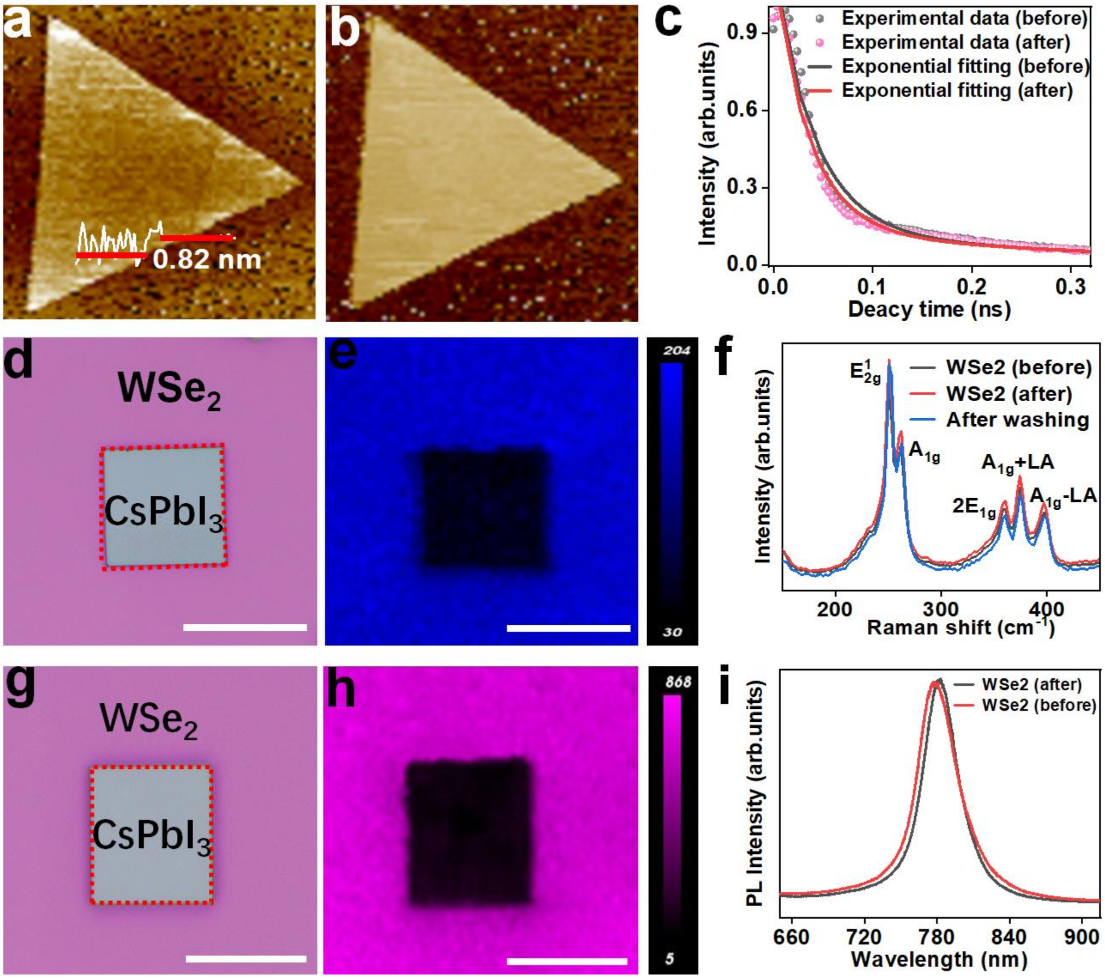

Supplementary Fig. 4. Monolayer nature of as-synthesized WSe_2 and its stability during $CsPbI_3$ epitaxy. a-b Atomic force microscope (AFM) image and phase image of as-synthesized WSe_2 on SiO_2/Si substrate. **c** Time-resolved photoluminescence (TRPL) experimental data and fitting results of WSe_2 before and after $CsPbI_3$ epitaxy. Raman mapping/spectra (**d-f**) and PL mapping/spectra (**g-i**) of WSe_2 at different conditions: before and after $CsPbI_3$ epitaxy and contacting region of $CsPbI_3$ after washing away $CsPbI_3$ using acetone. Scale bar, 15 µm. In Figure **(c), (f)** and **(i)**, the before and after are the abbreviations for "before $CsPbI_3$ epitaxy" and "after $CsPbI_3$ epitaxy", respectively.

Supplementary Table 1. Detailed fitting parameters of TRPL results of monolayer WSe_2 before and after $CsPbI_3$ epitaxial growth

Samples	A_1 (%)	τ_1 (ns)	A_2 (%)	τ_2 (ns)
WSe_2 (before)	0.90	0.041	0.10	0.34
WSe_2 (after)	0.87	0.036	0.13	0.33

Supplementary Fig. 5. Band structure determination

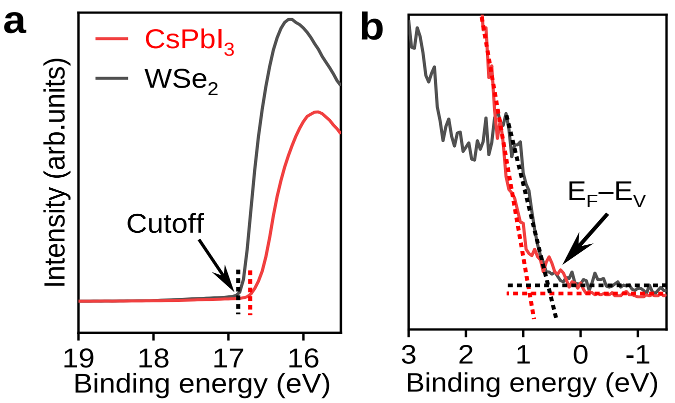

Supplementary Fig. 5. Ultraviolet Photoemission Spectroscopy (UPS). **a** Secondary electron cutoff of $CsPbI_3$ and WSe_2 films as measured by UPS; vertical dotted lines are located at the intersection between linear fits and mark the cutoff position. **b** Valence band photoemission spectra near the Fermi energy E_F with position of the valence band maximum pinned at the onset in the photoelectron signal as determined by linear fits.

Ultraviolet Photoemission Spectroscopy (UPS) was employed to determine the conduction and valence band positions of each semiconductor. Photoemission not only measures the binding energy (E_B) of valence electrons with respect to the chemical potential, but also provides a means for determining the work function (E_f) of the material which sets the minimum kinetic energy (E_K) required for photoelectrons to escape the film surface based on $E_K = h\nu - E_f - | E_B |$, where $h\nu$ is the photon energy [11]. Therefore, since E_K must be positive, a decrease in the photoelectron signal in the form of an intensity cutoff occurs at $|E_B| = h\nu - E_f$, which is able to read from the photoelectron traces obtained by using a He I-UV source ($h\nu$ = 21.22 eV).

Supplementary Fig. 5a-b show the cutoff energy region and the valence band region of the $CsPbI_3$ and WSe_2. Take the UPS spectrum of $CsPbI_3$ as an example for detailed analysis, the position of the electron affinity (Fermi level) versus vacuum is the difference between the high binding energy cutoff and the radiation energy of He I (21.22 eV).

16.70–21.22 = -4.52 eV

The low binding energy cutoff (0.98 eV) determines the position of valence band maximum (VBM). Therefore, the position of the VBM relative to the vacuum level is:

-4.52–0.98 = -5.50 eV

Considering the measured optical bandgap of ~1.76 eV obtained from PL spectrum or optical absorption. The conduction band minimum (CBM) is determined to be -3.64 eV. This also suggests that the Fermi level is closer to the CBM than to the VBM, and therefore the $CsPbI_3$ is n-type.

As a result, the $CsPbI_3$ and WSe_2 were demonstrated to be n- and p-type semiconductor, respectively. So, the interfacial bandgap alignment diagram is characterized by a type-II bandgap alignment, which imply that PL of each component in hybrid heterojunction can quench due to charge transfer-induced spatial separation

of an electron-hole pair in two adjacent layers, showing great agreement with PL experimental observations. Similarly, all other samples were analysed based on this protocol.

Supplementary Fig. 6-7. The chemical composition analysis of epitaxial $CsPbI_3$ flakes

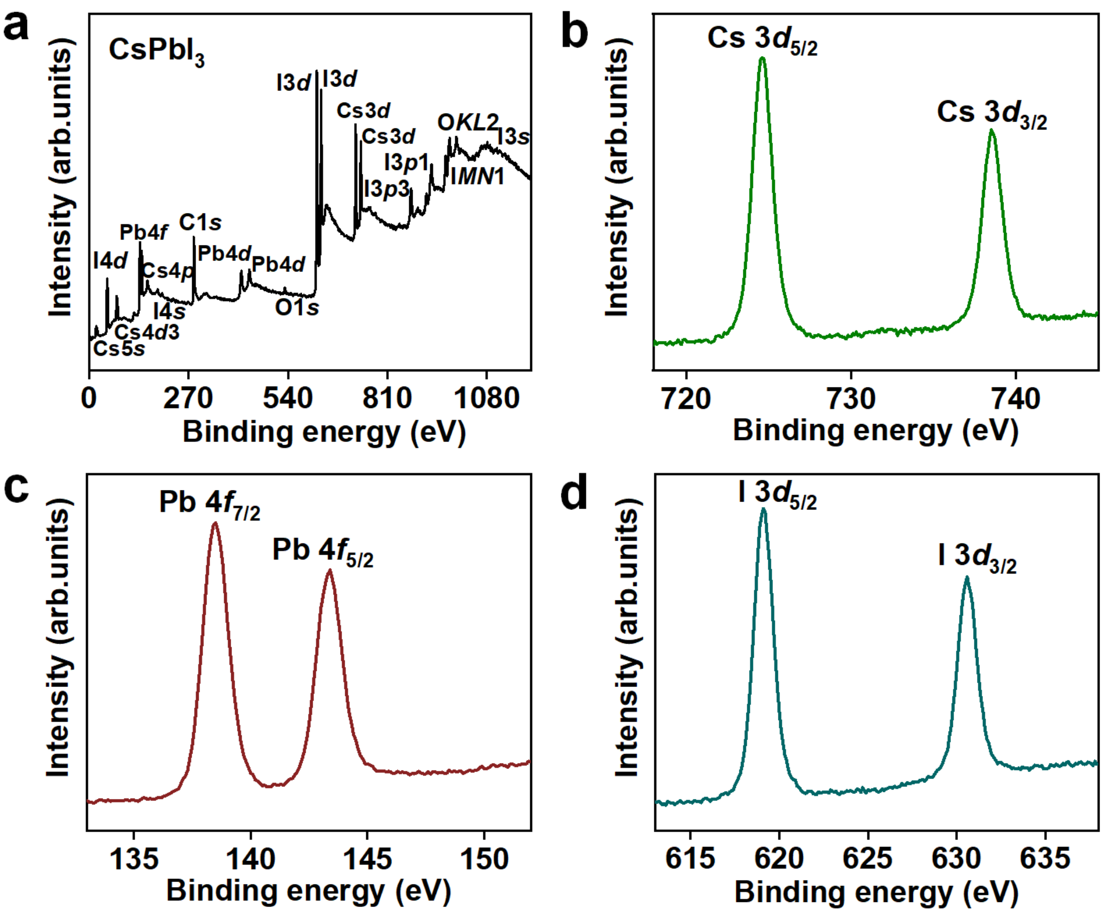

Supplementary Fig. 6. X-ray photoelectron spectra (XPS) of epitaxial $CsPbI_3$ flakes. a XPS survey of $CsPbI_3$. **b-d** XPS spectra of Cs, Pb and I.

The X-ray photoelectron spectroscopy (XPS) was applied to probe the chemical composition of epitaxial $CsPbI_3$ flakes. The survey scan XPS spectrum demonstrates high chemical purity of epitaxial $CsPbI_3$ perovskite (Supplementary Fig. 6a). The spectra of single Cs 3*d*, Pb 4*f* and I 3*d* were examined as shown in Supplementary Fig. 6b-d. It has been found that the couple peaks located at 724.4 and 738.3 eV are attributed to Cs 3$d_{5/2}$ and Cs 3$d_{3/2}$, while the strong peaks at around 138.6 and 143.3 eV come from Pb 4$f_{7/2}$ and Pb 4$f_{5/2}$, respectively. The prominent peaks of I 3$d_{5/2}$ and I 3$d_{3/2}$ of I 3*d* are located at around 618.9 and 630.5 eV, respectively. Those collected results of Cs 3*d*, Pb 4*f*, and I 3*d* are consistent with the reported values for the $CsPbI_3$ perovskites[12].

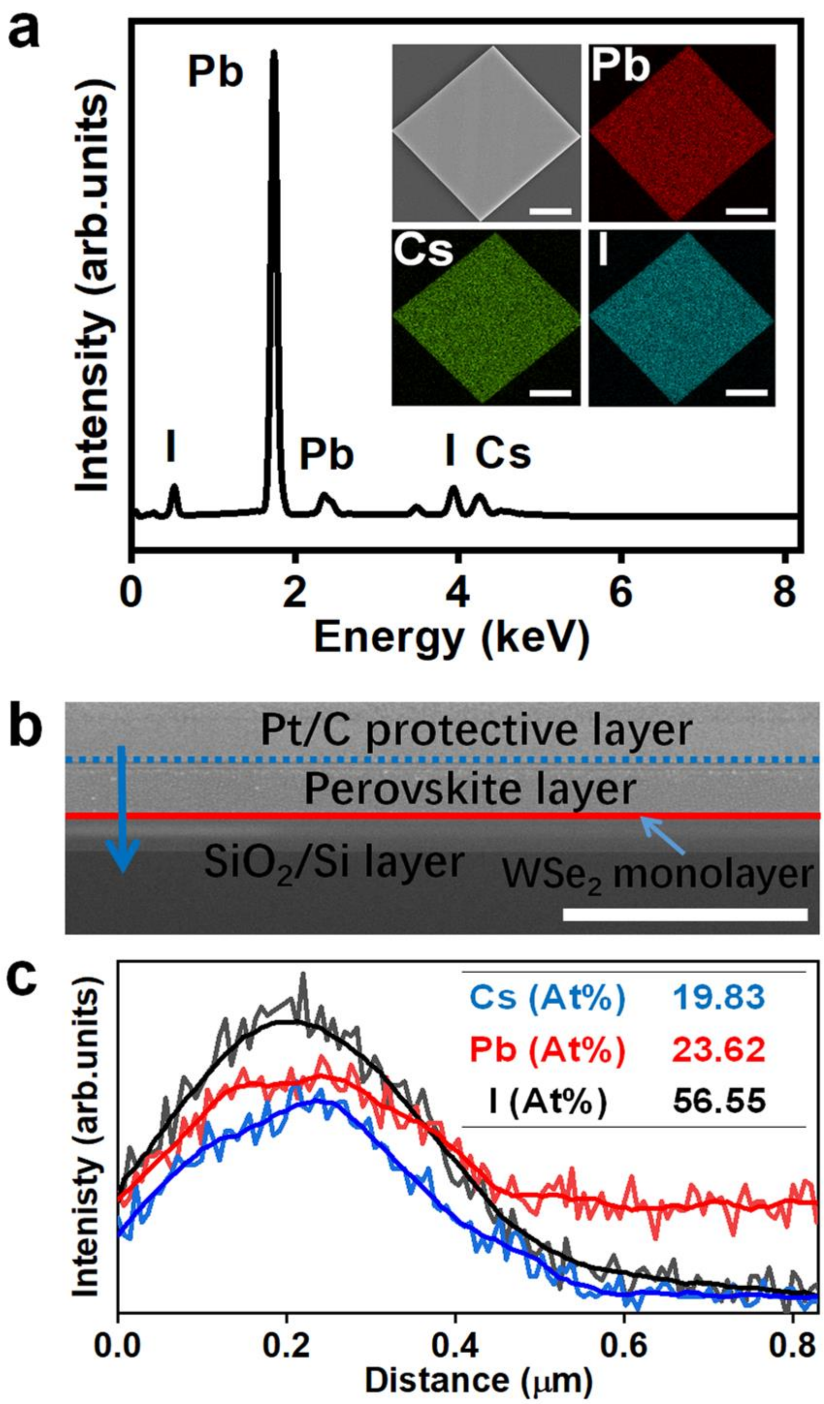

Supplementary Fig. 7. Spatial compositional characterization. a Energy dispersive spectroscopy (EDS) of epitaxial $CsPbI_3$. **b-c** Cross-sectional profile of epitaxial $CsPbI_3$ crystal and its line EDS.

To further characterize the compositional uniformity of epitaxial $CsPbI_3$, Energy dispersive spectroscopy (EDS) analysis was then performed to examine the elemental composition of the epitaxial perovskites. The EDS elemental mapping images show that all elements are uniformly dispersed in the square-shaped epitaxial $CsPbI_3$ (Supplementary Fig. 7a). The atomic ratio of Cs: Pb: I is 19.7%: 21.2%: 59.1%, consistent with the reasonable 1:1:3 stoichiometry ratio within the experimental error.

The cross-sectional sample was fabricated by focused ion beam. From the extracted interface, we can clearly observe that the uniform and ordered $CsPbI_3$ film without grain boundaries, demonstrating that the epitaxial monocrystalline $CsPbI_3$ renders a clean and sharp interface (marked in Supplementary Fig. 7b). The EDS line profile was recorded to reconfirm the composition uniformity, marked by blue row. Supplementary Figure 7c represents the EDS line profiles obtained for elements Cs, Pb and I across the junction, which exhibit nearly a stoichiometric ratio of 1: 1: 3 (19.83: 23.62: 56.55), showing excellent compositional uniformity in epitaxial $CsPbI_3$.

Supplementary Fig. 8. Phase identification of epitaxial $CsPbI_3$ flakes

To identify the perovskite polymorph of as-synthesized $CsPbI_3$, we conducted an elaborate and comparative analysis by combining the established X-ray diffraction (XRD) knowledge from literatures and experimental data. It is well-known that $CsPbI_3$ can exist in three distinct "black" perovskite phases (α, β, and γ phases), which all are characterized by corner-sharing octahedra but by subtle differences in the degree of symmetry, the corresponding structure models and XRD standard powder patterns are shown in Supplementary Fig. 8a-d. In term of crystal structure, the α phase belongs to cubic, consisting of prefect undistorted corner-sharing lead iodide octahedral, the β phase is a tetragonal distortion of the α phase, in which the corner-sharing lead iodide octahedra rotates and breaks the cubic symmetry. The γ phase comes from a second distortion leading to an orthorhombic structure with an additional tilt of the lead iodide octahedra. In addition, the δ phase is classified as yellow nonperovskite phase, featuring 1D double chain PbI_6^{4-} edge-sharing octahedral[13]. Accordingly, the XRD patterns also experience gradual evolution, reflected by the fact that the number of features present in the diffraction pattern increases with disorder (from the α to β to γ phase) and the characteristic perovskite peaks were observed for both β to γ phase, the difference is the peaks located near 15° and 30° 2θ (2-theta). Specifically, single strong characteristic perk (001) located at 13.8° 2θ emerge in ɑ-phase $CsPbI_3$, while peak splitting in this characteristic position of this splitting peak located at 14.18° and 14.36° for β and γ phase, respectively. Correspondingly, the other characteristic peaks also follow the similar splitting and shift to high angel side. For example, the (002) peak shifts from 27.7° to 28.59° to 28.97° 2θ, corresponding from α to β to γ phase, respectively[14].

As shown in Supplementary Fig. 9a-c, the characteristic peak near 15° of as-grown $CsPbI_3$ sample was splitted into two peaks, with one relatively strong peak position located at 14.72° 2θ (002) and another located at 14.89° (110). This trend is also present for the characteristic peak near 30° 2θ. Specifically, the relatively weak spitting peak at 28.6° emerges, the characteristic peak corresponding to (004) facet located at 29.1°. These results match well with the XRD standard powder pattern of γ phase, but with small difference existing. The peak intensity of (002) and (004) are stronger than the standard ones, which are likely attribute to the texture effect from preferential crystal facet growth and the single crystal nature of perovskite. A little peak position shift was also observed, which may be contributed by relatively large crystal size fabricated by vapor deposition route and released interface strains owing to dangling bound-free van der Waals substrate. In addition, a strong peak located near at 20°, which originates

from preferential orientation from the unique substrate symmetry-guided van der Waals epitaxy[13-15]. By combining the experimental data examination and theoretically comparative analysis, we can safely conclude that the epitaxial $CsPbI_3$ crystal belongs to orthorhombic (γ) phase (denoted as $CsPbI_3$ in this manuscript unless otherwise stated).

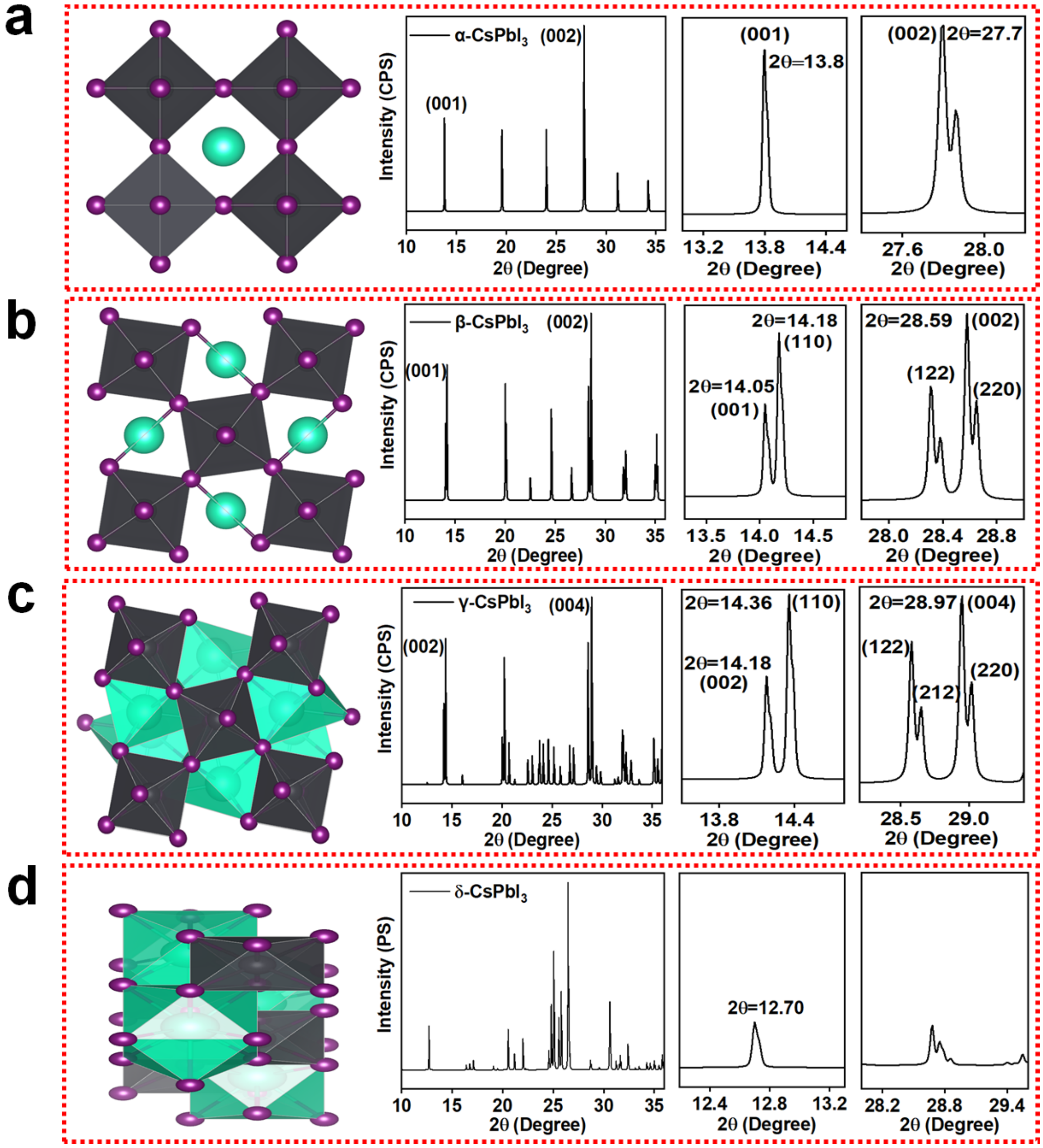

Supplementary Fig. 8. Theoretical crystal structures and corresponding X-ray diffraction (XRD) patterns. a-c Crystal structure model of three perovskite phase (α, β, and γ) with corner-sharing octahedra for $CsPbI_3$ and corresponding XRD standard powder patterns. **d** Crystal structure and XRD standard powder pattern of non-perovskite phase $CsPbI_3$ (δ phase). The characteristic peaks are enlarged for clear comparison. 2θ represents the 2-theta for all Figures.

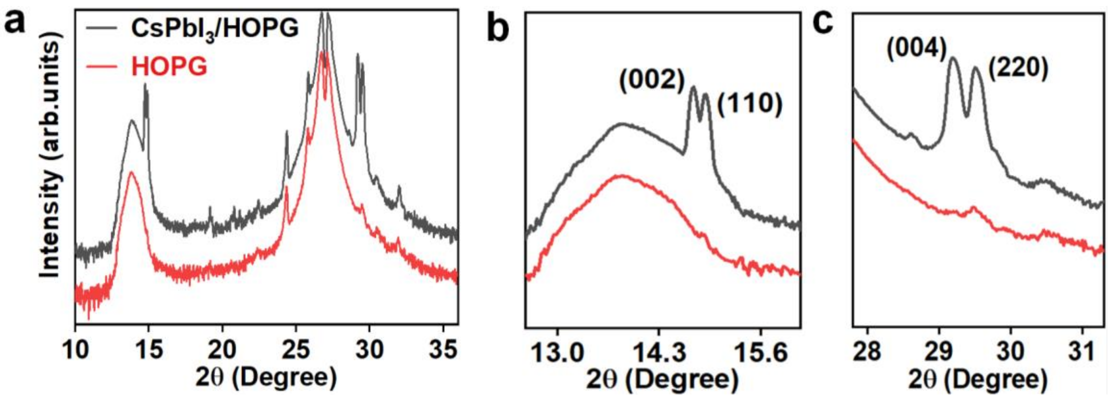

Supplementary Fig. 9. Experimental X-ray diffraction (XRD) results. a-c Experimental XRD pattern of epitaxial $CsPbI_3$ flakes and their magnified spectra. 2θ represents the 2-theta for all Figures.

Supplementary Fig. 10-11. Structures of monolayer WSe_2 and $CsPbI_3$ and interfacial structure models

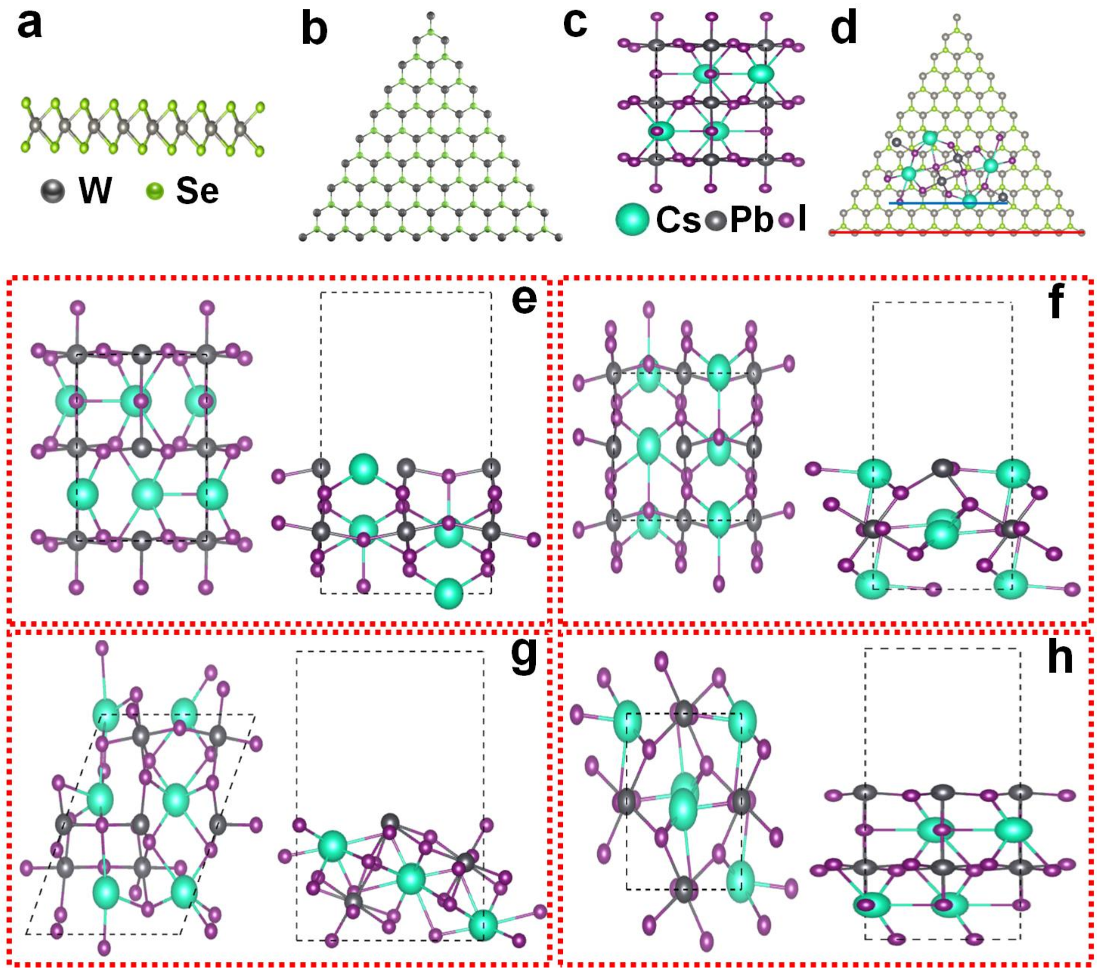

Supplementary Fig. 10. Atomic structures. **a-c** Atomic structures of monolayer WSe_2, $CsPbI_3$ perovskite, and $CsPbI_3/WSe_2$ heterostructure, where the in-plane rotation angle of $CsPbI_3$ on monolayer WSe_2 is defined as 0º **(d)**, and perovskite structures with (001) **(e)**, (100) **(f)**, (010) **(g)** and (111) **(h)** facets from top and side view.

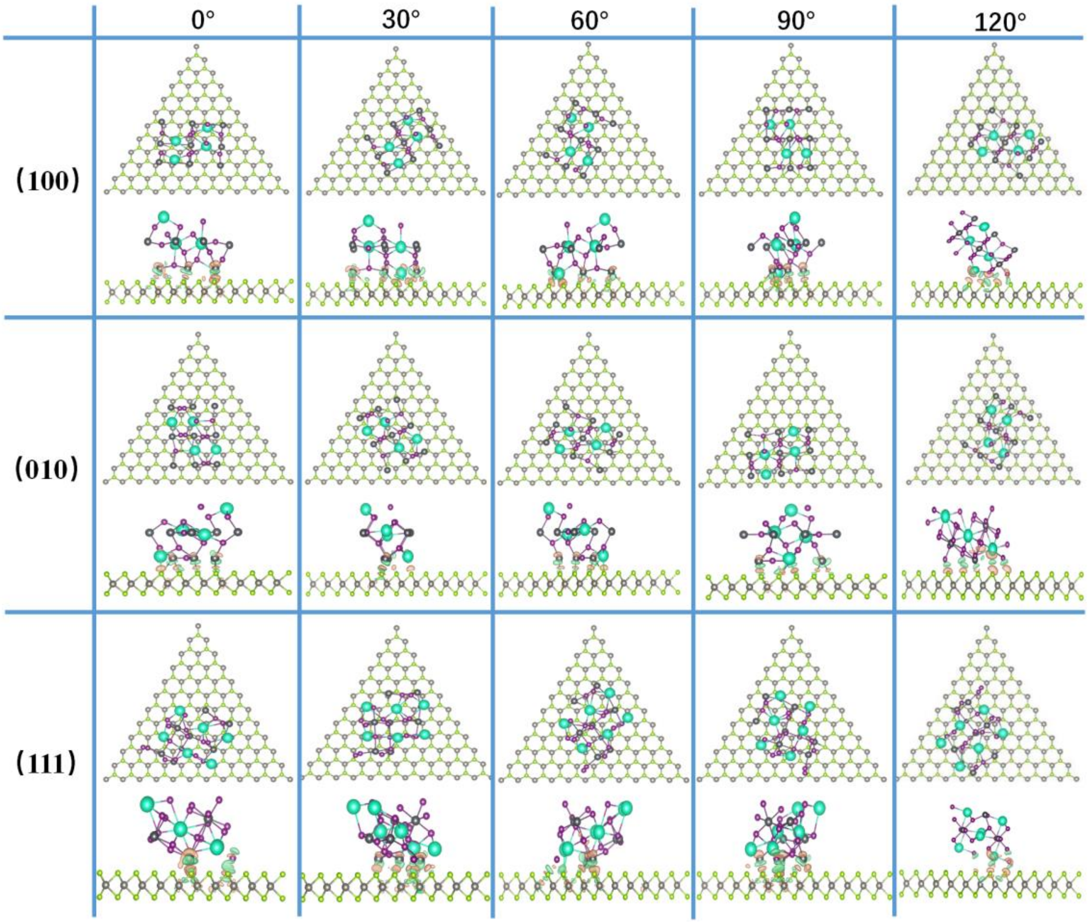

Supplementary Fig. 11. Interfacial configurations of $CsPbI_3/WSe_2$ heterostructure with different out-of-plane facets and in-plane orientations of epitaxial $CsPbI_3$.

Supplementary Fig. 12-13. Epitaxy-controlled nucleation and growth dynamics

The influences exerted by monolayer WSe_2 on $CsPbI_3$ epitaxial dynamics essentially originate from the unique surface and crystal structure features. At first, monolayer WSe_2 is a three-atom-thick 2D material with polarized atomic bonds, which will screen out the remote interactions from random fluctuant potential-field energy of underlying SiO_2 substrate, thus guaranteeing a robust environment for site-specific nucleation and homogeneous growth. Moreover, the dangling bond-free van der Waals surface of monolayer WSe_2 gives rise to a much lower adatom diffusion energy barrier and thus renders a much longer surface diffusion distance, which suggests that the relaxation of adatoms to energetic favorable site is not limited by kinetic barriers during epitaxy[16], an essential determiner contributing to searching correct lattice site and feeding nuclei proliferation. Furthermore, the coincidence lattice matching between WSe_2 and $CsPbI_3$ enables stable nuclei seeding with thermodynamically favorable orientation and facet, thus paving the well-defined epitaxial pathway and providing an additional knob for nanostructure design[17].

Benefiting from the robust and reproducible fabrication of monolayer WSe_2 and epitaxy of $CsPbI_3$, the growth dynamic evolution of $CsPbI_3$ epitaxy can be experimentally captured on the single particle level, thereby providing reasonable

rationales for application-oriented material design. In our experiments, the nucleation density, side length, growth rate and crystal morphology transformation were systematically investigated at $CsPbI_3$ epitaxy-accessible temperature window. The evolution of nucleation density as a function of growth temperature and corresponding optical images of resultant crystals were summarized in Supplementary Fig. 12a. At lower source temperature of 510 °C, the $CsPbI_3$ crystals nucleated at the level of 3120+430 mm^{-2}, with rectangle and orbicular (suborbicular) crystal domains coexisted. When increasing the source temperature to 515 °C and 520 °C, the nucleation density reduced to 1706+285 mm^{-2} and 896+202 mm^{-2}, respectively, with only rectangle crystal domains formed. The fitting results for temperature-dependent nucleation density demonstrated that the nucleation density exponentially decrease with temperature increase. Furthermore, the lateral size of epitaxial crystal may be tailored by controlling growth duration. For example, the side length of the $CsPbI_3$ domain is controlled to be 12.8 ± 2.95 μm, 20.4 ± 2.8 μm and 27.4 ± 3.03 μm within 2, 3 and 4 minutes (numbered by arithmetic average) at higher temperature (520 °C) (Supplementary Fig. 12c-e). The fitting results demonstrated that the growth rate follows the linear growth mode (the Pearson correlation coefficient of the linear fitting is 0.99), with a rate up to 167 nm s^{-1}, which is an order of magnitude larger than that of halide perovskite nanowires (4 nm s^{-1}) and films (1 μm min^{-1}) grown on mica[18]. In contrast, the growth rate at lower temperature (510 °C) undergo a different growth mode, which include an initial linear growth with a rate of about 101 nm s^{-1} and a sublinear growth with much slower rate, eventually harvesting a suborbicular crystal but still keeping expected epitaxial relationship, manifested by a well-defined orientation with the underlying WSe_2 edge (Supplementary Fig. 12b).

The edge-induced nucleation and growth behavior is common in 2D material-based heterostructure growth. The underlying growth mechanism lies in the nucleation and growth from the energy-favorable edge site of the monolayer WSe_2 [19]. Specifically, the edge of monolayer WSe_2 is characterized by Se/W-terminated dangling-bond states. These dangling bonds act as strong absorption sites for capturing precursor radicals (Cs^+ and $[PbI_6]^{4-}$). The adatoms can randomly nucleate and then grow at edge. However, the disparate dangling-bond densities of the limited edge of monolayer WSe_2 and SiO_2 surface leads to imbalanced mass transport surrounding the adjacent regions and non-uniform adatom deposition at the heterointerface between SiO_2 and WSe_2 edge. The large fluctuation and frequently local disequilibrium of mass transport at WSe_2 edge finally result in an intertwined random nucleation and anisotropic growth scenario. The outcome is the irregular crystals located on WSe_2 edge[20].

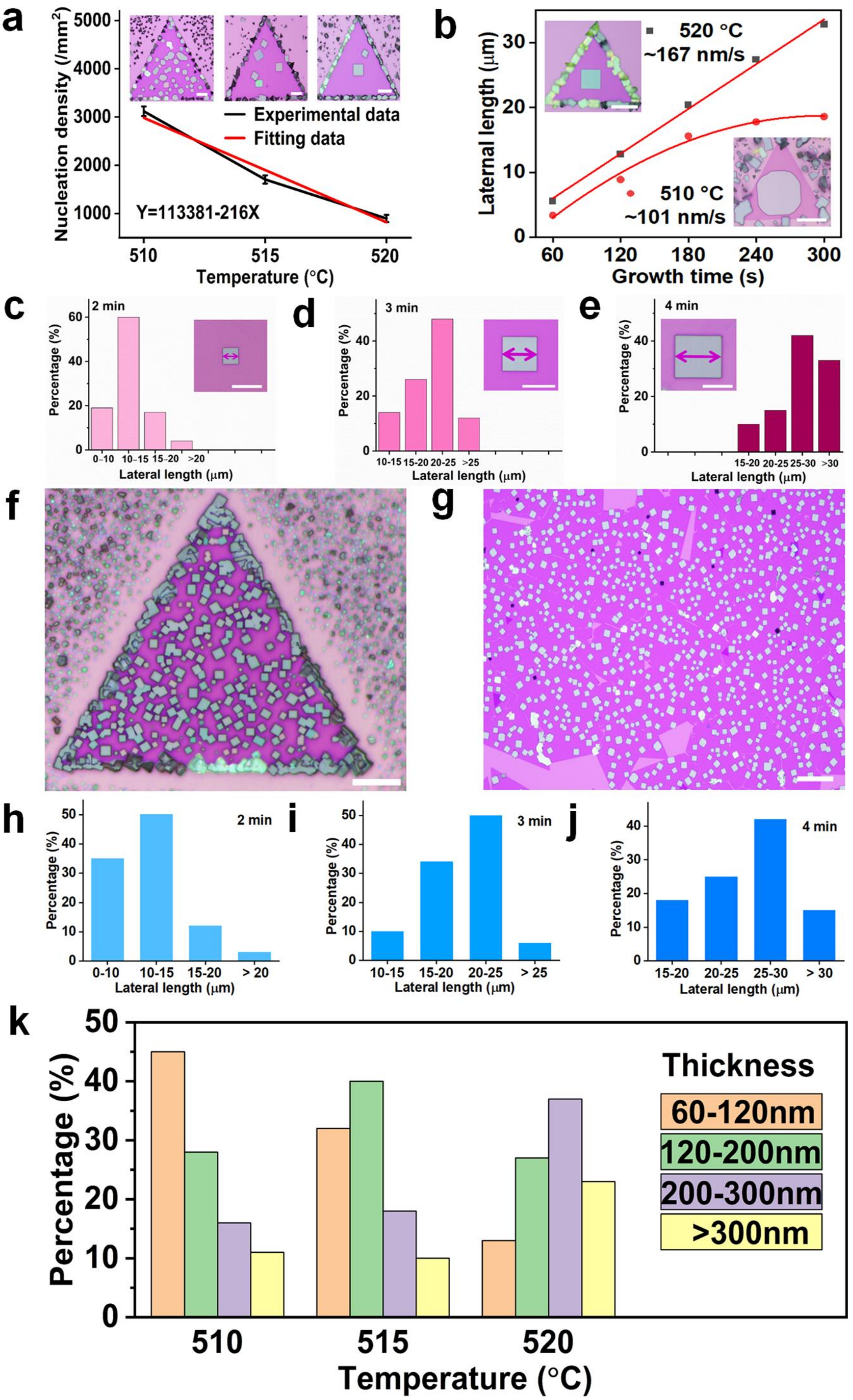
a
Nucleation density (/mm²)
Experimental data
Fitting data
Y=113381-216X
Temperature (°C)
b
Lateral length (μm)
520 °C
~167 nm/s
510 °C
~101 nm/s
Growth time (s)
c
2 min
d
3 min
e
4 min
Percentage (%)
Lateral length (μm)
f
g
h
2 min
i
3 min
j
4 min
k
Percentage (%)
Thickness
60-120nm
120-200nm
200-300nm
>300nm
Temperature (°C)

Supplementary Fig. 12. Experimental observations of epitaxial nucleation and growth evolution. **a** Epitaxial nucleation density as a function of growth temperature. **b** Crystal growth rate obtained by the edge length as a function of growth time relationship. The statistical distribution of the lateral width of $CsPbI_3$ plates obtained with growth times of 2 min **(c)**, 3 min **(d)** and 4 min **(e)**. To minimize the edge nucleation competition effect of WSe_2 monolayer, the large-area single crystal WSe_2 (edge length of about 300 μm in **(f)**, scale bar, 50 μm) and polycrystalline monolayer WSe_2 (edge length of as large as 700 μm in **(g)**, scale bar, 100 μm) are selected as growth templates for observing the lateral size distribution as a function of growth time. The statistical distribution of the lateral length of perovskite nanoplates obtained with growth times of 2 min **(h)**, 3 min **(i)** and 4 min **(j)** on large-area WSe_2 monolayer. **k** Temperature-dependent thickness evolution.

Whether the lateral size of epi-layer is limited by the underlying 2D materials mainly depends on the nucleation location. If the initial nucleation site is close to the edges of monolayer WSe_2, the lateral growth rate is suppressed. If the initial nucleation sites keep their certain distance from the edge of monolayer WSe_2, the lateral growth is impervious to the presence of the edge. In the former case, the dangling bond sites at the edge of the monolayer WSe_2 act as capturing sites to influence the mass transport and change the distribution of perovskite precursor ions in the growth process. The competitive depletion of precursor ions at edge thus suppresses the nourishment concentration for continuous growth. The characteristic distance is determined by diffusion-controlled length scale (l_d) based on the DDA (deposition, diffusion, and aggregation) model. In the growth model, $l_\mathrm{d} \approx (D/F)^{1/6}$, where l_d is the distance of the region where no deposition occurs, D is a diffusion constant, and F is a flux of monomer[21]. At the edge and/or perovskite nucleation at the edge, the adatoms are intrinsically attached to the active dangling bond states. D is thus very small and the length scale of depletion is rather limited to their neighboring region. Therefore, the nucleation far from the edge is free from the influence the edge of underlying materials.

To minimize the effect of edge states of monolayer semiconductor, we expand the surface area of 2D WSe_2 for growing the halide perovskites. The surface area of WSe_2 monolayer is as large as 7800 μm^2 and 2 mm^2 for single crystal domain and polycrystalline film, respectively. The experimental results are shown in Supplementary Fig. 12f and g. Although polycrystalline nature of large-area WSe_2 film, the halide perovskites still kept the facet and orientation growth selectivity, suggesting that the grain boundary has insignificant effect on lateral size of perovskite growth. We then make a statistical investigation on the lateral size distribution as a function of growth time. The number of investigated perovskite nanoplate is more than 600, which ensure enough sample quantity for obtaining the reliable results. The results presented in Supplementary Fig. 12h-j clearly show that the lateral length distribution follows the similar trends with ordinary size WSe_2 monolayer in Supplementary Fig. 12c-e. Specifically, the average lateral length of the perovskite domain is estimated to be 12.2 ± 1.85 μm, 22.4 ± 2.1 μm and 28.4 ± 2.15 μm within 2, 3 and 4 min. The lateral length within longer growth time (3 min and 4 min) is a bit larger than those observed

in Supplementary Fig. 12d and e. This optimized investigation demonstrated that halide perovskite grown at the edge of monolayer WSe_2 has a slight effect on the in-plane perovskite growth by competitive depletion of feeding precursor.

The in-plane epilayer growth on 2D semiconductor is a kinetically controlled process, where the growth temperature is the main influencing factor. In general, 2D substrates provide low diffusion barriers and adhesion energy to adatoms due to the dangling bond-free inert surface. Therefore, adatoms can quickly diffuse and add on to the fastest growth front of the growing epilayer to extend it. At high temperature, adatoms with high kinetic energy have the increased diffusion rate. Consequently, adatoms can easily and faster migrate to the growth edge for continuously feeding the in-plane epitaxial growth of epilayer. Additionally, the high local supersaturation gradient is synchronously introduced[22]. The single nucleation can receive sufficient nutrient atoms, thus accelerating the growth rate. When adatoms deposited on 2D material surface with insufficient thermal energy at lower temperature, limited adatoms can arrive at the growing front of nucleus, thus reducing the in-plane growth rate. It is should be noted that the growth temperature should be optimized for controlling the in-plane growth of epilayer. The reason is that the rate of absorption and deabsorption should be balanced owing to the much lower adsorption energy.

The out-of-plane growth of 3D perovskite is intrinsically driven by the non-van der Waals bonding nature of the 3D nonlayered perovskite. The ionic-bonding lattice networks lead to low cohesive energy of perovskites, which makes the out-of-plane growth more sensitive to growth temperature[23]. The out-of-plane growth are composed of two stages: new round of nucleation on the first perovskite layer and consequent growth along vertical direction. The vertical growth process is much more thermodynamically favorable. The temperature-dependent thickness evolution is investigated and shown in Supplementary Fig. 12k.

To decipher these epitaxy growth behaviors, the relationship between nucleation density of $CsPbI_3$ domains and the inverse temperature was modelled by using the Nernst–Einstein equation:$k = Ae^{-Ea/RT}$, where k is the constant of nucleation rate, which is proportionate to the nucleation density, A is a pre-exponential factor, Ea refers to activation energy for the nucleation reaction, R is the universal gas constant and the temperature is T (K as a unit), from which the extracted Ea is negative, demonstrating that nucleation density exponentially decreases with the temperature increase[24], which is in accordance with the experimental observations and fitting (Supplementary Fig. 13a). To acquire quantitative insights, the essential kinetic parameters of $CsPbI_3$ epitaxy on monolayer WSe_2 were probed by theoretical calculations, with which the diffusion energy barriers and diffusion paths (Supplementary Fig. 13b-c)) of $CsPbI_3$ on WSe_2 (001), $CsPbI_3$ (001) and the edge of $CsPbI_3/WSe_2$ are extracted to be 0.16 eV, 0.28 eV and 0.31 eV, respectively, and the corresponding adsorption energy (Supplementary Fig. 13e) are found to be -0.47 eV, -0.63 eV and -0.78 eV, respectively. According to the Fick's law, the surface diffusion length of an adatom (L) is defined by the mean free path of an adatom on the surface, which estimates the magnitude of the random walk it experiences before re-evaporation and can be described by the following equations (1)-(3)[23,25]:

$$L = \sqrt{D\tau_a} \quad (1)$$

$$D = 1/4\, a^2 v \exp(-E_d/KT) \quad (2)$$

$$\tau = v^{-1} \exp(-E_{\mathrm{ad}}/KT) \quad (3)$$

ere τ is lifetime of adatom, v is the vibration frequency and k is the Boltzmann constant, D is the surface diffusivity of adatom on the substrate surface, a is the single hop distance of adatom on monolayer WSe_2 surface. By solving the equations we can clearly see that the diffusion and adsorption energy conjointly contribute to the surface diffusion length of adatom, $L = 0.5a\exp((E_{\mathrm{ad}} - E_{\mathrm{d}})/2KT)$, which is exponentially temperature-dependent. The higher the temperature is, the longer the surface diffusion length is. On the basis of nucleation theory, the long diffusion distance means that, kinetic barrier of epitaxy on WSe_2 monolayer may not set restriction on the migration of adatoms to energetic favorable sites, which means large critical nuclei formation with reduced density to be achieved since simultaneous collisions of arriving adatoms on the surface are greatly suppressed. By comparison, amorphous SiO_2 surface is filled with abundant unsaturated bonds, which act as the anchors for easily capturing the incoming adatoms, eventually giving rise to limited diffusion distance. Under this circumstance, the high concentrated stable uncleus are most likely to form and are insensitive to temperature variations[25].

In the surface growth model, the position where adatoms could potentially incorporated into lattice for crystal growth will be the region around the already-formed stable nucleus within the width of L. Upon stable uncleus formation, the interaction on the nuclei (-0.63 eV) and edges of $CsPbI_3/WSe_2$ (-0.78 eV) established a favorable energetic landscape, leading to a preferential precursor adsorption and fast growth compared to the blank area (WSe_2 surface, -0.47 eV) and giving an interpretation on hanging growth across the underlying WSe_2 edge (Fig. 3f). In detail, as long as the stable nucleation formed on monolayer WSe_2, the oriented growth following the $CsPbI_3$ edge continued thermodynamically, which also radically demonstrated that the monolayer WSe_2 has extremely strong guiding ability in epitaxial halide perovskite. This growth behavior is largely different from the 2D materials epitaxy on sapphire and 2D material heterostructures growth[26,27]. As soon as the nucleus begin to grow, mass transport to the nucleus leads to the depletion of vaporized precursors (atoms) over the blank regions to the unsaturation points, eventually forming a concentration gradient as driving force to feed the fast growth of perovskite crystal, which can be demonstrated from the experimental observations that no precursor deposition surrounding crystal occurred. The higher and more uniform concentration gradient around the existing nuclei is, the faster and more linear the growth rate is. Consequently, in the sparse nucleation system, the single nucleation can receive sufficient nutrient atoms, thus accelerating the growth rate, and vice versa. As a result, random nucleation is inhibited and the accelerated growth is maintained in a linear fashion. The atomically sharp terrace steps of surface morphology characterized by atomic force microscope presented in Supplementary Fig. 13d and rectangular crystal island indicate that the heteroepitaxy of $CsPbI_3$ on monolayer WSe_2 is a hybrid layer-by-layer and Volmer-

Weber island growth mode, which possibly results from the facts that: the stronger adatom cohesive force of the overlayer material compared to the surface adhesive force between overlayer and substrate and the new atomic layer formation following 2D nucleation and growth mechanism[28].

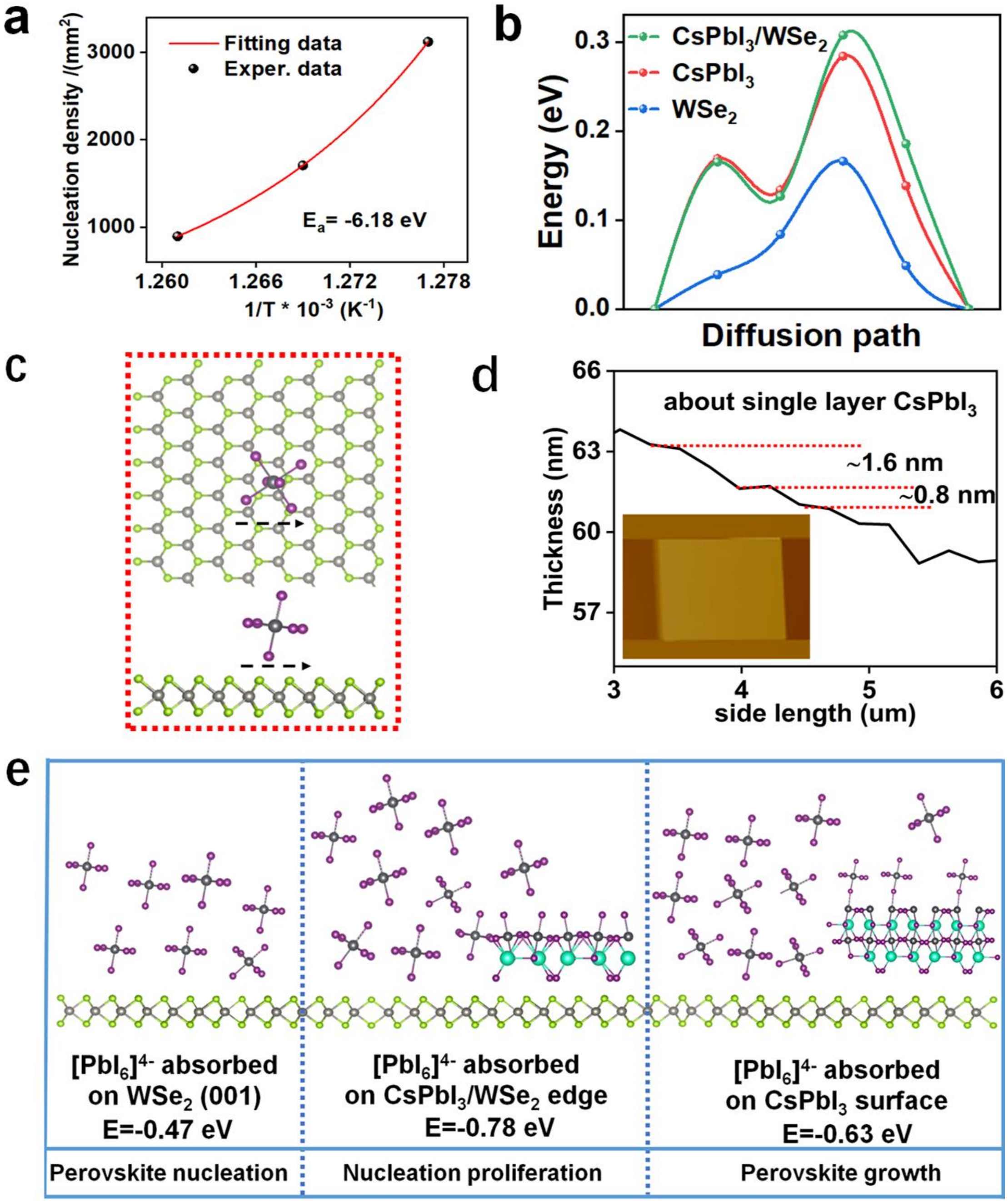

Supplementary Fig. 13. Theoretical understanding monolayer WSe_2 effect on epitaxial nucleation and growth dynamics. **a** Plotted Arrhenius curves of statistical nucleation density at different temperatures. **b** The calculated surface diffusion barriers for $CsPbI_3$ adatoms WSe_2 (001), $CsPbI_3/WSe_2$ edge and $CsPbI_3$ (001) surfaces. **c** The atomic models of simulated surface diffusion paths for perovskite molecules over WSe_2 layer. **d** the atomic morphology of epitaxial $CsPbI_3$. **e** In schematic, perovskite $[PbI_6]^{4-}$ octahedron sublattices were absorbed on WSe_2 (001) surface, $CsPbI_3/WSe_2$ edge and $CsPbI_3$ surface for describing the epitaxial nucleation and growth process, the Cs^+ is

omitted for clarity. It should be noted that at nucleation and growth process, absorbing precursors on $CsPbI_3/WSe_2$ and $CsPbI_3$ surface are concurrent.

To offer more contribution on the understanding of such hybrid heterostructure growth, we try our best to provide some suggestions on the growth controllability. In term of thickness, it should be tailored based on the specific optoelectronic applications. For light-harvesting optoelectronic (photovoltaic and photo-detection), the thickness should be controlled from 100 nm to 500 nm in order to maximize light absorption for improving efficiency and sensitivity. This can be simply controlled by growth temperature and time. For optical-addressable excitonic and quantum devices, the thickness should be controlled as thin as possible because ultrafast energy transfer, strong interfacial coupling and manageable exciton dynamics are much more favorable. This can be manipulated by space-confined growth method and simultaneous reduction of growth temperature and time. As for the location control of epi-layer, the strategies may include laser-defined defect-induced nucleation, hetero-nucleation-defining approach and pre-defining 2D material patterns. The location controllability is critical to fabricate heterostructure arrays, which is an important goal for practical optoelectronic applications.

Supplementary Fig. 14. Crystallinity quality characterization

To verify the crystallinity quality of the epitaxial perovskite, the cross-sectional high angle annular dark field scanning transmission electron microscope ((HAADF-STEM)) were performed. The cross-sectional specimen of $CsPbI_2Br/WSe_2$ heterostructure on SiO_2/Si substrate (Supplementary Fig. 14a) was prepared by focused ion beam (FIB). The resultant cross-sectional sample is shown in Supplementary Fig. 14b, in which the Pt and C layer are served as protective layer for minimizing the damage during FIB fabrication process. The cross-section of the halide perovskite displays a grain boundary-free and uniform/smooth morphology, indicating that as-grown halide perovskite belongs to a typical single crystal domain. The corresponding selected area electron diffraction (SAED) pattern presented in Supplementary Fig. 14c shows a single set of separated diffraction spots, strongly confirming that the epitaxial perovskite is a single crystal. More importantly, the atomic-resolution HAADF-STEM observations at low-magnification (Supplementary Fig. 14d) and high-magnification (Supplementary Fig. 14e) show that our epitaxial perovskite single crystal have very high crystalline quality without defects and vacancies even over a large area. The zoomed-in image (inset of Fig. 14e) reveals a nearly perfect periodic atom arrangement. Specifically, the significantly periodic high-resolution TEM image shows light gray and white atomic spots due to their small and large atomic numbers. The bright white atom is attributed to Cs, which occupy the site between the sub-lattice octahedral frameworks. Based on the difference of atomic volume, the Cs, Pb, I and Br are well-arranged into an octahedral framework. These direct images convincingly prove the high crystallinity quality of our heterostructures. Furthermore, the clearly resolved lattices spacing of 0.58 nm corresponds to the (001) plane of perovskite lattices, which is well consistent with the SAED result. The corresponding cross-sectional energy dispersive

spectrometer (EDS) elemental mapping images (Supplementary Fig. 14f) clearly display the spatial distribution of the element Cs, Pb, I and Br, whose uniform color contrasts further confirm the compositional homogeneity and high crystallinity quality.

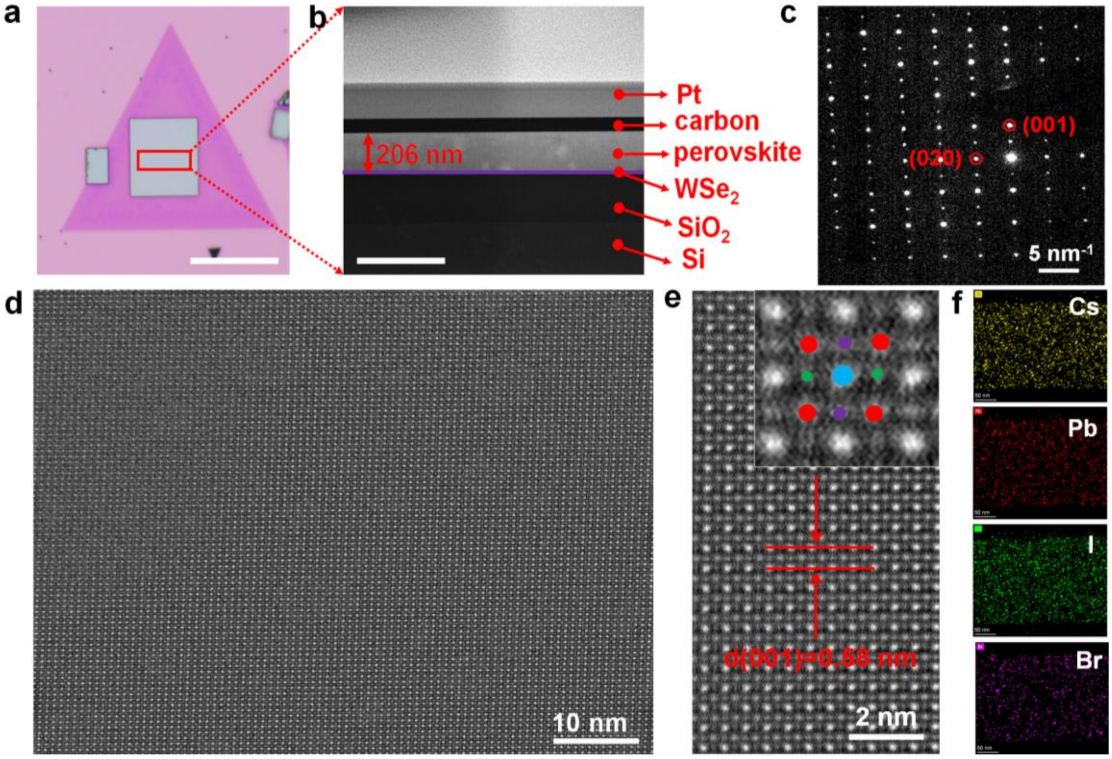

Supplementary Fig. 14. Crystallinity quality characterization by cross-sectional high-angle annular dark field scanning transmission electron microscope (HAADF-STEM). a The optical image of $CsPbI_2Br/WSe_2$ heterostructure (scale bar: 15 μm), where perovskite is well aligned with the edge of the monolayer WSe_2 and is used to prepare the cross-sectional specimen for HAADF-STEM characterization. Due to the soft nature of perovskite lattices, the focused ion beam in the process of fine ion-beam milling is set to be as low as possible to minimize the structural damage. The prepared cross-sectional sample is shown in (**b**). Scale bar, 500 nm. **c** The corresponding selected area electron diffraction (SAED) pattern of the cross-sectional sample in (**b**). **d-e** Low-magnification and high-magnification HAADF-STEM images of the inner area of the perovskite domain. Cs: blue, Pb: red, I: purple, Br: green. **f** Energy dispersive spectrometry (EDS) mapping images of Cs, Pb, I and Br from the cross-sectional sample of $CsPbI_2Br/WSe_2$ heterostructure.

Supplementary Fig. 15. Optical-gain lifetime

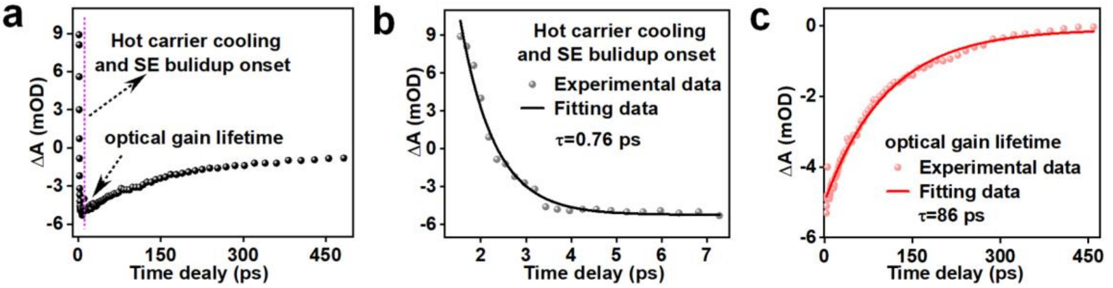

Supplementary Fig. 15. The estimation of optical gain lifetime. **a** The kinetics of stimulated emission feature in $CsPbI_2Br/WSe_2$ heterostructure at 660 nm above lasing threshold. **b-c** The magnified kinetics for hot-carrier cooling and SE buildup onset with timescale of 0.76 ps and gain decay lifetime of 86 ps, fitted by the exponential decay function[29].

Supplementary Fig. 16. The coherence property of emission

Optical coherence is the predictability of laser wave properties (amplitude and phase) from the process of stimulated emission, due to the generation of photons with "copied" wave traits (phase, wavelength, polarization, and propagating direction) from its spontaneously emitted "seed" photon. Laser light has two kinds of optical coherence, temporal coherence and spatial coherence[30,31].

Temporal coherence corresponds to the preservation of the phase relationship with time. The necessary conditions for temporal coherence are that all photons should be emitted with same phase and should have the same wavelength. Therefore, spectral narrowing is a basic indicator of the temporal coherence of lasing emission and is usually used as a measure of the degree of coherence [32,33]. Supplementary Figure 16 shows the comparison of the photoluminescence (PL, Supplementary Fig. 16a), amplified spontaneous emission (ASE, Supplementary Fig. 16b) and single-mode lasing (Supplementary Fig. 16c) of $CsPbI_2Br/WSe_2$ heterostructure. The PL displays a broad and featureless spectrum with full width at half maximum (FWHM) of 32.5 nm, ASE shows a relative narrower spectrum with FWHM of 8.3 nm, while the lasing peak exhibit a very narrow single spectrum with FWHM of 0.7 nm. The monochromatic linewidth below 1.0 nm is well consistent with the emission characteristic of halide perovskite laser, which also suggests that the output light is temporally coherent[34]. The observed linewidth narrowing is also in agreement with the Schawlow–Townes equations, which theoretically predict a sharp decrease of linewidth at the transition from incoherent to coherent emission[32,33]. To further quantitatively assess the degree of the temporal coherence, we estimate the corresponding coherent time (T_c) and length (L_c) based on the equation (4)-(5)[35,36].

$$L_c = cT_c \tag{4}$$

$$L_c = \lambda^2/\Delta\lambda \tag{5}$$

Where the c is the speed of light, λ is the wavelength of lasing peak and $\Delta\lambda$ is the linewidth of the lasing spectrum. The estimated coherent length is as long as 622 μm,

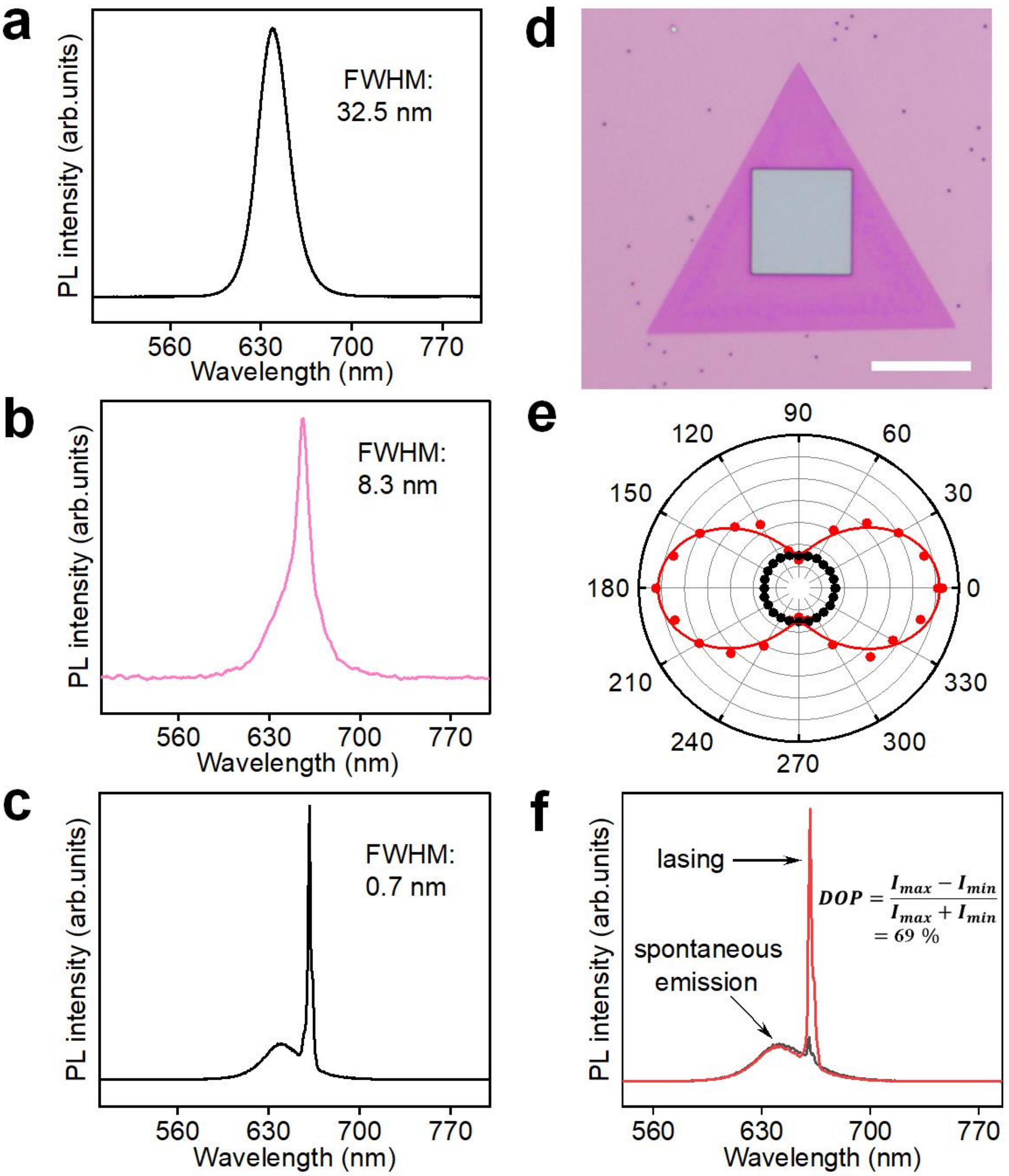

Supplementary Fig. 16. The optical coherence property of lasing action in $CsPbI_2Br/WSe_2$ heterostructure laser. a-c A comparison of the photoluminescence (PL), amplified spontaneous emission (ASE) and single mode lasing of $CsPbI_2Br/WSe_2$ heterostructure, respectively. **d** The optical image of $CsPbI_2Br/WSe_2$ heterostructure for polarization angle-dependent lasing action. Scale bar: 15 μm. **e** Intensity polar plot of $CsPbI_2Br/WSe_2$ lasing output through a rotational analyzer. The experimental data for single mode lasing and spontaneous emission are marked by red dots and black dots, respectively. The solid lines are fits to Malus's law. **f** Lasing spectra of a single $CsPbI_2Br/WSe_2$ heterostructure pumped above lasing threshold with orthogonal detection polarizations. The detected polarization characteristics demonstrated a linear polarization with degree of polarization (DOP) of 69%, showing a strong output polarization and thus long-range spatial coherence.

which is more than an order of magnitude and six times longer than the state-of-art reports on halide perovskite lasers (20 μm[37] and 115.6 μm[38]), respectively. The corresponding coherent time is 0. 21 ps. The high temporal coherence performance of epitaxial heterostructure microlaser can be attributed to self-organized high-quality whispering-gallery-mode (WGM) semiconductor resonator, minimized energetic disorder landscape and high optical gain property.

Spatial coherence measures the correlation of wave phases extending along a single wavefront, which depends upon the transverse mode discrimination property of the laser resonator. This feature is typically characterized by a well-defined beam with well-defined phase across it and overall beam directionality[30,39]. The experimental observation in Fig. 7d in manuscript clearly shows four corner bright lasing output beams when the pump density exceeds the threshold, clearly suggesting the existence of spatial coherence[30]. To estimate the degree of the spatial coherence, the beam directionality emitted by our heterostructures laser (Supplementary Fig. 16d) is assessed by the linear polarization of the lasing emission, as shown in Supplementary Fig. 16e and f. The degree of polarization (DOP = $(I_{\mathrm{max}} - I_{\mathrm{min}})/(I_{\mathrm{max}} + I_{\mathrm{min}})$, where I_{max} and I_{min} are the maximum lasing intensity and the minimum lasing intensity, respectively), of the single-mode lasing was measured up to 69 %, significantly higher than that of the spontaneous emission peak (~4%). Coupled with the lasing spectra with only one mode, the single lasing mode could be assigned to the fundamental transverse Gaussian beam mode (TEM_{00})[34,38]. The robust polarization selectivity in the WGM cavity suggests good spatial coherence. The reasons could be attributed to the following reasons. (1) In self-organized perovskite laser, short- and long-range electron-hole exchange interactions result in a splitting of the band-edge excitonic states into an optically inactive singlet state and three optically active triplet states. The competition between three-fold degenerate bright-triplet and a dark singlet transitions leads to the linear-polarised outputs[40]. (2) The optical birefringence from Vernier-effect coupling forces single-mode outputs with strong linear polarization properties[37]. The good coherence deuced from notable linewidth narrowing also rule out the possibility of localized excitons as a source of lasing[41]. If possible, the higher disorder states in individual perovskite laser should render a lower threshold than that of heterostructure laser, which is inconsistent with our experimental observations. Furthermore, the main factors governed the coherence of laser and improvement methods are discussed accordingly based on the theory of the linewidth of semiconductor lasers[42,43]. In general, the width of the laser linewidth mainly originates from the fluctuations in the phase of the optical field. On the one hand, these fluctuations arise from spontaneous emission events, which discontinuously alter the phase and intensity of the lasing field. On the other hand, the change in refractive index due to the change of carrier density can results in an additional phase shift of the laser field and in additional line broadening. The unified theory for qualifying the linewidth (Δf) is expressed as following equation (6)[44]:

$$\Delta f = \frac{u_{\mathrm{g}}^2 hvgn_{\mathrm{s}}\alpha_{\mathrm{m}}(1+\alpha^2)}{8\pi P_0} \tag{6}$$

where u_{g} is the group velocity, hv is the energy of the output laser, g is the modal gain,

n_s is referred to as the spontaneous emission factor, α_m is the facet loss factor, $1 + \alpha^2$ is enhancement factor, P_0 is the output power. Based on the equation, three strategies can be inspired to compress the linewidth and thus enhance the coherence[31]. Firstly, reducing cavity loss caused by facet and waveguide losses, which can be achieved by embedding the semiconductor nanostructures into other ultra-low loss optical cavities such as silica whispering-gallery-mode cavities or distributed Bragg reflector cavities. Secondly, increasing the photon lifetime within the cavity can suppresses intensity fluctuations of the emission, enhancing the second-order coherence. This can be realized by tailoring the spatial structures of lasing modes and their nonlinear interactions within the gain material. Finally, enhancing the output power might be viable path to regulate the coherence by integrating an external cavity for underpinning the feedback modal.

The sharp linewidth narrowing, monochromatic lasing output, long-range coherence, high-bright lasing output beam and large output polarization unambiguously demonstrate coherent lasing emission.

Supplementary Table 2. Comparison of perovskite WGM lasers

Among the comparative perovskite WGM lasing action, the key parameters, S, D and M represent single-mode, double-mode and multimode lasing, respectively.

Materials	Pump source	λ(nm)	Threshold (μJ/cm^2)	Lasing mode	Ref.
$MAPbI_3$ nanoplates	400nm, 150 fs, 1 kHz	786	128	M	[45]
$CsPbBr_3$ QD film	400nm, 100 fs, 1kHz	524.5	11.6K	M	[46]
$MAPbBr_3$ microdisk	400nm, 120 fs, 1kHz	557.5	3.6	D	[47]
$CsPbBr_3$ nanoplate	400nm, 150 fs, 295kHz	545	38	M	[48]
$MAPbBr_3$ microdisk	400nm, 150 fs, 1kHz	560	10.2	M	[49]
$MAPbI_3$ nanoplatelet	400nm, 50 fs, 1kHz	785	37	M	[50]
$CsPbX_3$ nanoplates	400 nm, 50 fs, 1kHz	680	10	M	[33]
$MAPbI_3$ thin film	645nm, 120 fs, 250 kHz	791.5	19.6	M	[51]
$MAPbBr_3$ microplates	400 nm, 150 fs, 1kHz	551.8	15.9	M	[52]
$FAPbI_3$ nanoplates	402 nm, 150 fs, 250 kHz	837	25	S	[53]
$MAPbI_3$	630 nm, 120	785	7.1	M	[54]

microdiscs	fs, 250 kHz				
$CsPbBr_3$ microdisk	450 nm, 7 ns, 10 Hz	700	200	M	[55]
$CsPbI_3$ microdisk-hBN	532 nm, 4.5 ns, 1.15 kHz	726	130	S	[56]
$(OA)_2PbBr_4$ microplate	400nm, 150 fs, 1 kHz	545	7.8	M	[57]
$MAPbX_3$ microplates	400nm,100 fs, 1 kHz	780	30	M	[58]
$CsPbBr_3$ microsphere	800 nm, 40 fs, 1 kHz	542.6	203.7	S	[59]
$MAPbBr_3$ sub-circular	450 nm, 100 fs, 1 kHz	550	6.4	M	[60]
$FAPbBr_3$ microdisks	400 nm, 100 fs, 1 kHz	552.9	70	S	[61]
$CsPb_2Br_5$ microplates	800 nm, 35 fs, 1 kHz	539.9	180	M	[62]
$CsPbBr_3$ microcrystal	490 nm, 25 ps, 1–10 Hz	532	65.5	S	[63]
$CsPbBr_3$ microplate	800 nm, 35 fs, 1 kHz	536.6	34.2	M	[64]
$CsPbI_2Br$-WSe_2	400 nm, 100 fs, 1 kHz	662	1.98	S	this work

Supplementary Fig. 17. Statistical lasing threshold distribution

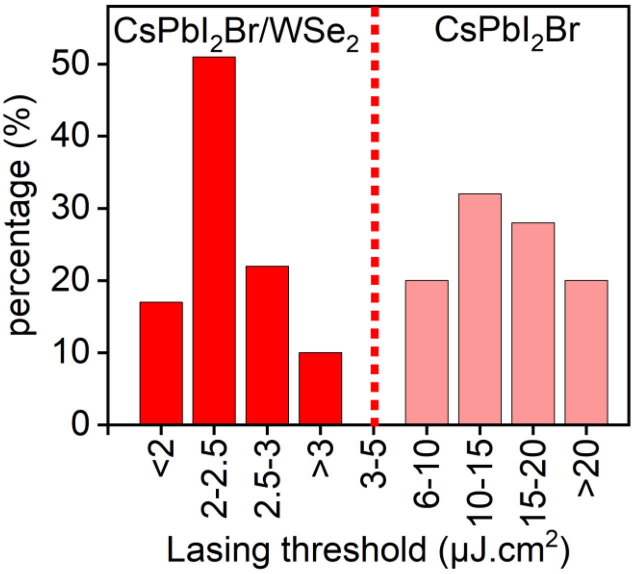

Supplementary Fig. 17. Statistical lasing threshold distribution of $CsPbI_2Br/WSe_2$ heterostructure and $CsPbI_2Br$ nanoplate. The selected $CsPbI_2Br/WSe_2$ heterostructure and $CsPbI_2Br$ nanoplat for examining the lasing threshold distribution feature thickness spanning from 170-210 nm and edge length ranging from 9 μm to 18 μm. This selection rule is based on the extensive experimental observations, where the smaller/larger and thinner perovskite lasers is hardly to lase. This is attributed to (1) the smaller edge length of perovskite laser suffer from inadequate optical gain that is unable to compensate the absorption loss, but too larger perovskite laser is incapable of forming high optical confinement for effective optical feedback; (2) the optical fields are well-confined inside the cavities and totally reflected between the polygonal facets to establish WGM waveguides[45,61]. With decreasing nanoplatelet thickness, the effective index of the photonic modes decreases, yielding a higher radiative loss or even the cutoff of the photonic modes.

Supplementary Fig. 18. Stability of perovskite lasers

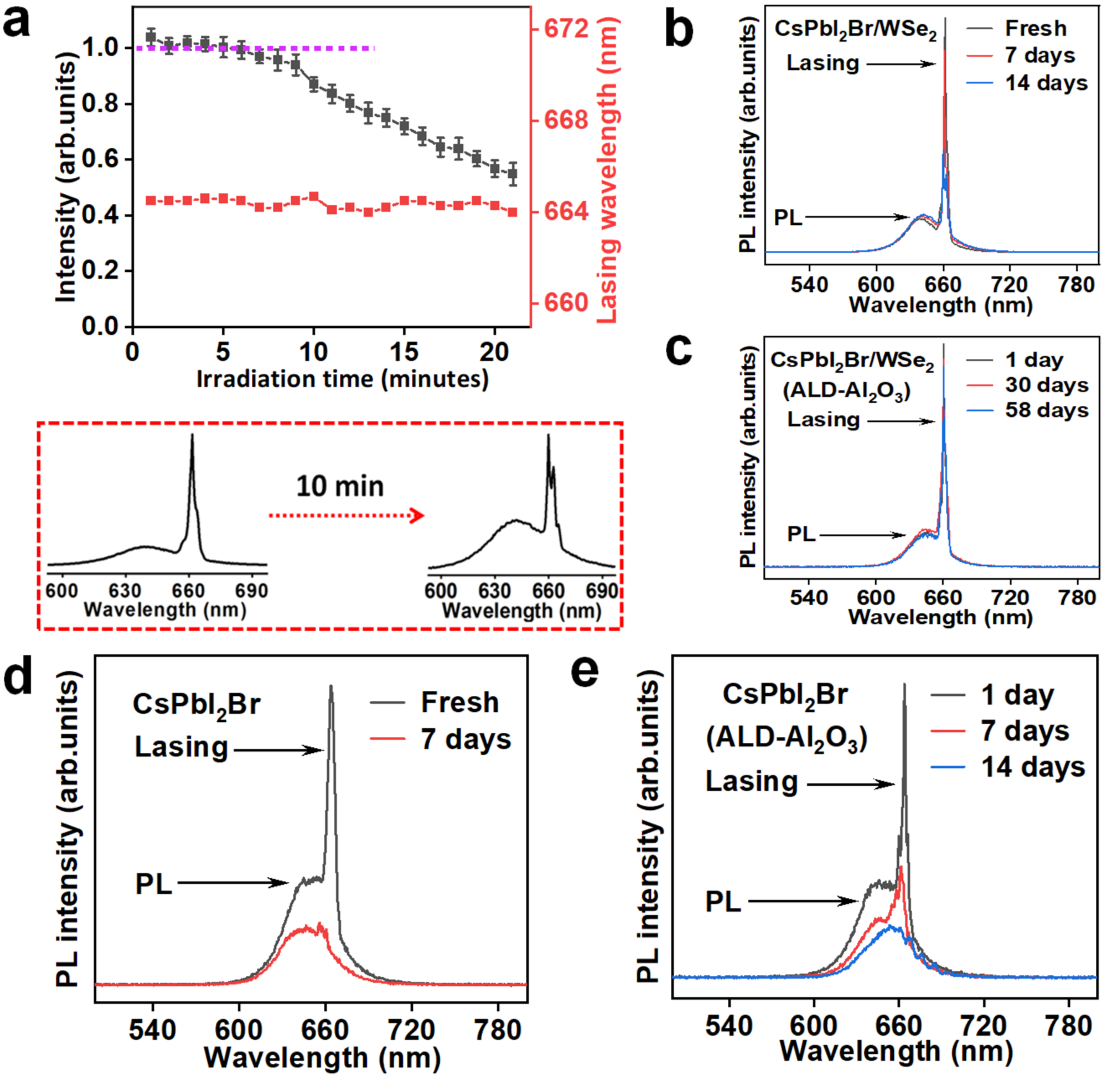

Supplementary Fig. 18. Stability of perovskite lasers. **a** Photostability of $CsPbI_2Br$ nanoplate grown on SiO_2/Si substrate, which shows average lasing stability lifetime of about 10 minutes. **b** Storage stability of $CsPbI_2Br/WSe_2$ heterostructure within air-filled tube. **c** Stability of $CsPbI_2Br/WSe_2$ heterostructure using large-scale on-chip microprocess-compatible atomic layer deposition (ALD) Al_2O_3 (10 nm) to conformally encapsulate the lasing medium. **d** Storage stability of $CsPbI_2Br$ nanoplate within air-filled tube. **e** Stability of $CsPbI_2Br/WSe_2$ heterostructure using large-scale on-chip microprocess-compatible ALD-Al_2O_3 (10 nm) to conformally encapsulate the lasing medium. PL: Photoluminescence.

The photostability of lasing of $CsPbI_2Br$ nanoplate (Supplementary Fig. 18a, top), assessed at the same irradiation conditions of $CsPbI_2Br/WSe_2$ heterostructure (Fig. 7l), is about 10 minutes, which is lower than that of $CsPbI_2Br/WSe_2$ heterostructure. The distinct change of lasing intensity after 10 minutes laser irradiation is presented in Supplementary Fig. 18a, bottom. In addition, the decay rate of lasing intensity of $CsPbI_2Br$ nanoplate is also faster than that of $CsPbI_2Br/WSe_2$ heterostructure. The

lasing position of $CsPbI_2Br$ nanoplate centers at 664.2 nm, and is subjected to irradiation time-dependent fluctuation, which is caused by light-induced Frenkel defect pair formation. The $CsPbI_2Br/WSe_2$ heterostructure can maintain lasing ability for 14 days (Supplementary Fig. 18b), but the lasing ability of $CsPbI_2Br$ nanoplate faded within 7 days storage, with a broad PL signal alone (Supplementary Fig. 18d). To improve the stability of perovskite lasers, we used large-scale on-chip microprocess-compatible atomic layer deposition (ALD) Al_2O_3 (10 nm) to conformally encapsulate the lasing medium. As expected, the stability of perovskite lasers greatly improved. The $CsPbI_2Br/WSe_2$ heterostructure maintained its lasing ability up to 58 days (Supplementary Fig. 18c), which is longer than that of $CsPbI_2Br$ nanoplate (about 7-14 days, Supplementary Fig. 18e). These results clearly demonstrated that the stability of $CsPbI_2Br/WSe_2$ heterostructure laser is enhanced due to the reduced energetic disorders in epitaxial perovskite lattices.

Supplementary Fig. 19. Lasing thresholds of $CsPbI_3/WSe_2$ (WS_2) heterostructures and $CsPbI_3$ nanoplates

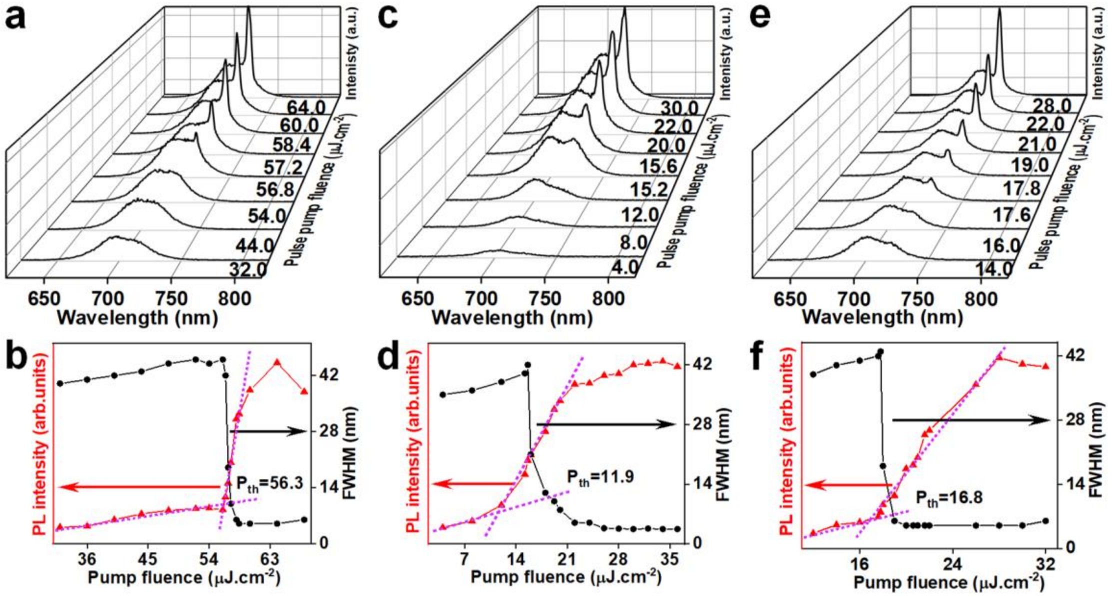

Supplementary Fig. 19. The lasing behaviors of $CsPbI_3/WSe_2$ (WS_2) heterostructures and $CsPbI_3$ naoplate. The pump fluence-dependent PL spectra and intensity/FWHM plots of $CsPbI_3$ naoplate **(a, b)** and $CsPbI_3/WSe_2$ heterostructure **(c, d)** and $CsPbI_3/WS_2$ heterostructures **(e, f).** The lasing threshold of $CsPbI_3$ nanoplate is 56.3 μJ cm^{-2}, which is far higher than that of $CsPbI_3/WSe_2$ and $CsPbI_3/WS_2$ heterostructures (11.9 μJ cm^{-2} and 16.8 μJ cm^{-2}, respectively).

Supplementary Note 1. Calculation of surface recombination velocity, diffusion coefficient

For monocrystalline perovskite film, the defects or trap states mainly gather on the surface, which have detrimental effects on semiconductor property, stability and device performances [65]. The surface-sensitive ultrafast transient reflection (TR) spectroscop is thus performed to quantitatively identify the surface luminous and nonluminous species

and provide detailed insight into the surface charge carrier dynamics, avoiding interferential signals from the bulk and interface. For a semiconductor, the complex refractive index is generally expressed as n+ik, where the real part (n) is much larger than the imaginary part (k), the reflectance change ΔR/R is mostly due to changes in n, which can be related to the optical absorption change due to band filling, charge carrier screening, band-renormalization, and free-carrier absorption. Under excited carrier density lower than 10^{18} cm^{-3}, we can take ΔR/R as proportional to carrier population in the near surface region [66,67]. The representative 2D pseudocolor TR spectra of the $CsPbI_2Br/WSe_2$ and $CsPbI_2Br$ are shown in Fig. 5g, h, the corresponding reflectance change (ΔR/R) kinetics extracted from the corresponding spectra are plotted in Fig. 5i.

The TR decay is attributed to surface recombination and carrier diffusion from the surface into the bulk. The former is associated with the surface defect concentration, while the latter is mainly dictated by the carrier concentration gradient. To obtain the corresponding surface recombination velocity (S) and carrier diffusion coefficient (D), the diffusion-surface-recombination model is adopted and the equation (7) is as followed [66-68]:

$$\frac{\partial N(\chi,t)}{\partial t} = D\frac{\partial^2(\chi,t)}{\partial \chi^2} - \frac{N(\chi,t)}{\tau_{bulk}} \quad (7)$$

where τ_{bulk} is the bulk lifetime, χ is the distance away from surface. The initial carrier distribution is given by equation (8):

$$\mathrm{N}(\chi,0) = N_0 \mathrm{e}^{-\alpha\chi} \quad (8)$$

where α is the absorption coefficient at the excitation wavelength. For our as-grown single crystals, the boundary conditions are defined as:

when χ=0, $\frac{\partial N(\chi,t)}{\partial \chi} = \frac{S}{D}N(0,t)$

and $\mathrm{N}(L,t) = 0$

where L is the thickness of the single crystal. Therefore, the normalized carrier density (N) as a function of time (t) and distance away from surface (χ) can be solved:

$$\mathrm{N}(\chi,t) = \frac{1}{2}exp\left(-\frac{\chi^2}{4Dt}\right)\left[w\left(\alpha\sqrt{Dt}-\frac{\chi}{2\sqrt{Dt}}\right)-\frac{S+\alpha D}{S-\alpha D}w\left(\alpha\sqrt{Dt}+\frac{\chi}{2\sqrt{Dt}}\right)+\frac{2S}{S-\alpha D}w\left(S\sqrt{\frac{t}{D}}+\frac{2\chi}{2\sqrt{Dt}}\right)\right]\exp\left(-\frac{t}{\tau_{\mathrm{bulk}}}\right) \quad (9)$$

Where $w_{(\chi)}$ is the complex error function with $\mathrm{w}(\chi) \equiv \exp(\chi^2)[1-ref(\chi)]$, the $\exp(-t/\tau_{bulk})$ value can be approximated as 1 because the bulk carrier lifetime (on ns timescale) of the single crystal is much longer than the probe time windom of the TR spectrum (on ps timescale). The carrier density in the probe region can be approximated as the carrier density at the surface, $N(0, t)$, because the carrier distribution is nearly flat in this regime.

Then, the S and D values can be obtained by fitting $N(0, t)$ using a universal global optimization approach. The produced results are S = 1300 cm s^{-1} and D = 0.52 cm^2 s^{-1}

for $CsPbI_2Br/WSe_2$, S = 3700 cm s^{-1} and D = 0.43 cm^2 s^{-1} for $CsPbI_2Br$. This large difference in S clearly indicates that the reduced defect density in WSe_2-enabled epitaxial growth; the small difference in D may stem from the similar low carrier concentration gradient under weak excitation fluence (initial carrier density is about 1.2x10^{17} cm^{-3}). Those results demonstrated that less disorder landscape is preferred in the epitaxial perovskite lattices.

Supplementary Note 2. Calculation of spontaneous emission coupling factor β

For the investigated $CsPbI_2Br$ nanoplate, the estimated volume of exciton generated in the $CsPbI_2Br$ nanoplate is as followed: V_C = 12.5 μm × 12.8 μm × 0.21 μm = 3.36 × 10^{-11} cm^3; the cavity quality factor Q is 1103; the mode peak wavelength is approximately 660 nm with a refractive index of about 2.3. Then the Purcell factor can be estimated to be F_p = 5.9 × 10^{-2} according to the following equation (10) [64,69]:

$$F_{\mathrm{p}} = \frac{3}{4\pi^2}\frac{Q}{V_C/(\lambda/n)^3} \tag{10}$$

And thus, the spontaneous emission coupling factor β can be estimated to be 5.5 × 10^{-2} based on the approximation equation (11):

$$\beta = \frac{F_{\mathrm{p}}}{1+F_{\mathrm{p}}} \tag{11}$$

Supplementary Note 3. Relationship between lasing peak position and Urbach tail

In polar semiconductors, the structural disorder from defects, impurities or phonons can induce an extension of the density of state into the band gap near the main edges of the bands, and optical transitions assisted by this extension of state give rise to an exponential tail near the fundamental absorption edge at the long wavelength direction (Urbach tail)[70]. The impact of Urbach tail on PL/lasing peak position relies on self-absorption of band-edge emission. This photophysic process can be described by the fundamental absorption rule of semiconductors when taking into consideration the contributions from the Urbach tail according to the equation (12)[45,71,72].

$$\alpha_{(E)} = \begin{cases} A_0\sqrt{\frac{K_BT}{2\sigma}}\exp\left[\frac{\sigma}{K_BT}(E-E_{\mathrm{cr}})\right] & E \geq E_g \\ A_0\exp\left[\frac{\sigma}{K_{\mathrm{B}}T}(E-E_g)\right] \text{ (Urbach rule)} & E < E_g \end{cases} \tag{12}$$

where A_0 is a constant, Eg is the optical band gap energy, k_BT is the thermal energy at room temperature (25.6 meV), σ is a dimensionless phenomenological parameter, the energy $E = h\nu$ with h being the Planck's constant, ν is the photon frequency, and Ecr = Eg + $k_BT/2\sigma$ is the crossover energy.

In solid materials, σ is defined as (equation (13))[70,73]:

$$\alpha = \alpha_0\frac{2KT}{h\omega_0}\tan h\left[\frac{h\omega_0}{2KT}\right] \tag{13}$$

with α_0 and ω_0 as constants. According to the Lambert-Beer law, the absorption coefficient $\alpha(E)$ in semiconductor can be defined by the following equation (14):

$$I_{(x)} = I_0 e^{-\alpha(E)x} \tag{14}$$

where I_0 and $I_{(x)}$ are the optical intensities at the zero and x position, respectively. Therefore, we get the PL/lasing peak spectrum at the position x based on the equation (15).

$$\mathrm{P}(x) = P_0 e^{-\alpha(E)x} \tag{15}$$

where S_0 is the initial spectrum (zero position). According to the Urbach theory, α_0 is variable with the inherent structure, and it is an indicator of the degree of disorder of crystalline materials. The higher the degree of local disorder, the smaller the σ value, thus more redshift of PL/lasing peak.

Supplementary References:

1 Kerner, R. A., Xu, Z., Larson, B. W. & Rand, B. P. The role of halide oxidation in perovskite halide phase separation. *Joule* **5**, 2273-2295 (2021).

2 Zhang, Z. et al. Robust epitaxial growth of two-dimensional heterostructures, multiheterostructures, and superlattices. *Science* **357**, 788-792 (2017).

3 Zhang, Z. et al. Endoepitaxial growth of monolayer mosaic heterostructures. *Nat. Nanotechnol.* **17**, 493-499 (2022).

4 Zhao, B. et al. High-order superlattices by rolling up van der Waals heterostructures. *Nature* **591**, 385-390 (2021).

5 Li, H. et al. Mechanical exfoliation and characterization of single- and few-layer nanosheets of WSe2, TaS2 , and TaSe2. *Small* **9**, 1974-1981 (2013).

6 Kim, H. et al. Synthetic WSe_2 monolayers with high photoluminescence quantum yield. *Sci. Adv.* **5**, eaau4728 (2019).

7 Li, S. et al. Vapour–liquid–solid growth of monolayer MoS_2 nanoribbons. *Nat. Mater.* **17**, 535-542 (2018).

8 Li, S. et al. Halide-assisted atmospheric pressure growth of large WSe_2 and WS_2 monolayer crystals. *Appl. Mater. Today* **1**, 60-66 (2015).

9 Ji, Q. et al. Revealing the Br&nsted-Evans-Polanyi relation in halide-activated fast MoS_2 growth toward millimeter-sized 2D crystals. *Sci. Adv.* **7**, eabj3274 (2021).

10 Li, X. et al. Surfactant-mediated growth and patterning of atomically thin transition metal dichalcogenides. *ACS Nano* **14**, 6570-6581 (2020).

11 Comin, R. et al. Structural, optical, and electronic studies of wide-bandgap lead halide perovskites. *J. Mater. Chem. C* **3**, 8839-8843 (2015).

12 Zheng, Z. et al. Space-confined synthesis of 2D all-Inorganic $CsPbI_3$ perovskite nanosheets for multiphoton-pumped lasing. *Adv. Opt. Mater.* **6**, 1800879 (2018).

13 Wang, K. et al. All-inorganic cesium lead iodide perovskite solar cells with stabilized efficiency beyond 15. *Nat. Commun.* **9**, 4544 (2018).

14 Marronnier, A. et al. Anharmonicity and disorder in the black phases of cesium

lead iodide used for stable inorganic perovskite solar cells. *ACS Nano* **12**, 3477-3486 (2018).
15 Sutton, R. J. et al. Cubic or orthorhombic? Revealing the crystal structure of metastable black-phase $CsPbI_3$ by theory and experiment. *ACS Energy Lett.* **3**, 1787-1794 (2018).
16 Kong, W. et al. Polarity governs atomic interaction through two-dimensional materials. *Nat. Mater.* **17**, 999-1004 (2018).
17 Wang, L., King, I., Chen, P., Bates, M. & Lunt, R. R. Epitaxial and quasiepitaxial growth of halide perovskites: New routes to high end optoelectronics. *APL Mater.* **8**, 100904 (2020).
18 Gao, M. et al. The making of a reconfigurable semiconductor with a soft ionic lattice. *Matter* **4**, 3874-3896 (2021).
19 Zhang, Z. et al. Epitaxial growth of two-dimensional metal–semiconductor transition-metal dichalcogenide vertical stacks (VSe_2/MX_2) and their band alignments. *ACS Nano* **13**, 885-893 (2018).
20 Markov, I. Influence of surface active species on kinetics of epitaxial nucleation and growth. *Mater. Chem. Phys.* **49**, 93-104 (1997).
21 Gu, Z., Huang, Z., Li, C., Li, M. & Song, Y. A general printing approach for scalable growth of perovskite single-crystal films. *Sci. Adv.* **4**, eaat2390 (2018).
22 Jiang, J. et al. Carrier lifetime enhancement in halide perovskite via remote epitaxy. *Nat. Commun.* **10**, 4145 (2019).
23 Guesnay, Q., Sahli, F., Ballif, C. & Jeangros, Q. Vapor deposition of metal halide perovskite thin films: Process control strategies to shape layer properties. *APL Mater.* **9**, 100703 (2021).
24 Zhang, S. et al. High-performance electronics and optoelectronics of monolayer tungsten diselenide full film from pre-seeding strategy. *InfoMat* **3**, 1455-1469 (2021).
25 Wang, Y., Shi, Y., Xin, G., Lian, J. & Shi, J. Two-dimensional van der Waals epitaxy kinetics in a three-dimensional perovskite halide. *Cryst. Growth Des.* **15**, 4741-4749 (2015).
26 Zhang, B. et al. Two-dimensional wedge-shaped magnetic EuS: Insight into the substrate step-guided epitaxial synthesis on sapphire. *J. Am. Chem. Soc.* **144**, 19758-19769 (2022).
27 Zhang, K. et al. Visualizing van der Waals epitaxial growth of 2D heterostructures. *Adv. Mater.* **33**, e2105079 (2021).
28 Zhao, L. et al. Vapor-phase incommensurate heteroepitaxy of oriented single-crystal CsPbBr3 on GaN: Toward integrated optoelectronic applications. *ACS Nano* **13**, 10085-10094 (2019).
29 Liu, Z. et al. Robust subwavelength single-mode perovskite nanocuboid laser. *ACS Nano* **12**, 5923-5931 (2018).
30 Samuel, I. D. W., Namdas, E. B. & Turnbull, G. A. How to recognize lasing. *Nat. Photonics* **3**, 546-549 (2009).
31 Cao, H., Chriki, R., Bittner, S., Friesem, A. A. & Davidson, N. Complex lasers with controllable coherence. *Nat. Rev. Phys.* **1**, 156-168 (2019).

32 Elbaz, A. et al. Ultra-low-threshold continuous-wave and pulsed lasing in tensile-strained GeSn alloys. *Nat. Photonics* **14**, 375-382 (2020).
33 Zhang, Q. et al. High-quality whispering-gllery-mode lasing from cesium lead halide perovskite nanoplatelets. *Adv. Funct. Mater.* **26**, 6238-6245 (2016).
34 Tay, Y. K. E., He, H., Tian, X., Li, M. & Sum, T. C. *Halide Perovskite Lasers* 1-19 (Springer Nature Singapore, 2022).
35 Svelto, O. *Principles of Lasers* 475-504 (Springer US, 2010).
36 Svelto, O. et al. *Springer Handbook of Lasers and Optics* 583-936 (Springer New York, 2007).
37 Song, J. et al. Continuous-wave pumped perovskite lasers with device area below 1 μm2. *Adv. Mater.* **35**, 2302170 (2023).
38 Huang, S. et al. Water-resistant subwavelength perovskite lasing from transparent silica-based nanocavity. *Adv. Mater.* **35**, 2306102 (2023).
39 Kozlov, V. G., Bulović, V., Burrows, P. E. & Forrest, S. R. Laser action in organic semiconductor waveguide and double-heterostructure devices. *Nature* **389**, 362-364 (1997).
40 Ye, J. et al. Direct linearly polarized electroluminescence from perovskite nanoplatelet superlattices. *Nat. Photonics* https://doi.org/10.1038/s41566-024-01398-y (2024).
41 Paik, E. Y. et al. Interlayer exciton laser of extended spatial coherence in atomically thin heterostructures. *Nature* **576**, 80-84 (2019).
42 Liu, Y. et al. Room temperature nanocavity laser with interlayer excitons in 2D heterostructures. *Sci. Adv.* **5**, eaav4506 (2019).
43 Reeves, L., Wang, Y. & Krauss, T. F. 2D material microcavity light emitters: To lase or not to lase? *Adv. Opt. Mater.* **6** 1800272 (2018).
44 Henry, C. H. Theory of the linewidth of semiconductor lasers. *IEEE J. Quantum Electron.* **18**, 259-264 (1982).
45 Zhang, Q., Ha, S. T., Liu, X., Sum, T. C. & Xiong, Q. Room-temperature near-infrared high-Q perovskite whispering-gallery planar nanolasers. *Nano Lett.* **14**, 5995-6001 (2014).
46 Wang, Y. et al. All-inorganic colloidal perovskite quantum dots: A new class of lasing materials with favorable characteristics. *Adv. Mater.* **27**, 7101-7108 (2015).
47 Liao, Q. et al. Perovskite microdisk microlasers self-assembled from solution. *Adv. Mater.* **27**, 3405-3410 (2015).
48 Zhang, H., Liao, Q., Wang, X., Yao, J. & Fu, H. Water-resistant perovskite polygonal microdisks laser in flexible photonics devices. *Adv. Opt. Mater.* **4**, 1718-1725 (2016).
49 Liu, X. et al. Periodic organic–inorganic halide perovskite microplatelet arrays on silicon substrates for room-temperature lasing. *Adv. Sci.* **3**, 1600137 (2016).
50 Wang, K. et al. Unidirectional lasing emissions from $CH_3NH_3PbBr_3$ perovskite microdisks. *ACS Photonics* **3**, 1125-1130 (2016).
51 Cegielski, P. J. et al. Integrated perovskite lasers on a silicon nitride waveguide platform by cost-effective high throughput fabrication. *Opt. Express* **25**, 13199-

13206 (2017).
52 Gu, Z. et al. Direct-writing multifunctional perovskite single crystal arrays by inkjet printing. *Small* **13**, 1603217 (2016).
53 Fu, Y. et al. Stabilization of the metastable lead iodide perovskite phase via surface functionalization. *Nano Lett.* **17**, 4405-4414 (2017).
54 Cegielski, P. J. et al. Monolithically integrated perovskite semiconductor lasers on silicon photonic chips by scalable top-down fabrication. *Nano Lett.* **18**, 6915-6923 (2018).
55 Lin, C. H. et al. Large-area lasing and multicolor perovskite quantum dot patterns. *Adv. Opt. Mater.* **6**, 1800474 (2018).
56 Yu, H. et al. Waterproof perovskite-hexagonal boron nitride hybrid nanolasers with low lasing thresholds and high operating temperature. *ACS Photonics* **5**, 4520-4528 (2018).
57 Li, M. et al. Enhanced exciton and photon confinement in Ruddlesden–Popper perovskite microplatelets for highly stable low-threshold polarized lasing. *Adv. Mater.* **30**, 1707235 (2018).
58 Huang, C. et al. Formation of lead halide perovskite based plasmonic nanolasers and nanolaser arrays by tailoring the substrate. *ACS Nano* **12**, 3865-3874 (2018).
59 Tang, B. et al. Ultrahigh quality upconverted single-mode lasing in cesium lead bromide spherical microcavity. *Adv. Opt. Mater.* **6**, 1800391 (2018).
60 Li, B. et al. Temperature dependent geometry in perovskite microcrystals for whispering gallery and Fabry–Pérot mode lasing. *J. Mater. Chem. C* **7**, 4102-4108 (2019).
61 Li, X. et al. Stable whispering gallery mode lasing from solution-processed formamidinium lead bromide perovskite microdisks. *Adv. Opt. Mater.* **8**, 2000030 (2020).
62 Liu, Z. et al. Mode selection and high-quality upconversion lasing from perovskite $CsPb_2Br_5$ microplates. *Photonics Res.* **8**, A31-A38 (2020).
63 Sun, X. et al. Lasing from solution-processed $CsPbBr_3$ octahedral resonators. *J. Lumin.* **229**, 117713 (2021).
64 Weng, G. et al. Electron–hole plasma lasing dynamics in $CsPbClmBr_{3-m}$ microplate Lasers. *ACS Photonics* **8**, 787-797 (2020).
65 Ni, Z. et al. Resolving spatial and energetic distributions of trap states in metal halide perovskite solar cells. *Science* **367**, 1352-1358 (2020).
66 Zhu, H. et al. Organic cations might not be essential to the remarkable properties of band edge carriers in lead halide perovskites. *Adv. Mater.* **29**, 1603072 (2016).
67 Yang, Y. et al. Top and bottom surfaces limit carrier lifetime in lead iodide perovskite films. *Nat. Energy* **2**, 16207 (2017).
68 Chen, W. et al. Highly bright and stable single-crystal perovskite light-emitting diodes. *Nat. Photonics* **17**, 401-407 (2023).
69 Wu, S. et al. Monolayer semiconductor nanocavity lasers with ultralow thresholds. *Nature* **520**, 69-72 (2015).
70 Van Mieghem, P. Theory of band tails in heavily doped semiconductors. *Rev. Mod. Phys.* **64**, 755-793 (1992).

71 Liu, X., Zhang, Q., Xiong, Q. & Sum, T. C. Tailoring the lasing modes in semiconductor nanowire cavities using intrinsic self-absorption. *Nano Lett.* **13**, 1080-1085 (2013).
72 Feng, L. et al. Constructing Urbach-tail-free and low-threshold perovskite heteronanowire lasers toward all-optical switching. *ACS Photonics* **9**, 459-465 (2022).
73 John, S., Soukoulis, C., Cohen, M. H. & Economou, E. N. Theory of electron band tails and the Urbach optical-absorption edge. *Phys. Rev. Lett.* **57**, 1777-1780 (1986).
